# Temporal Expression and Localization Patterns of Variant Surface Antigens in Clinical *Plasmodium falciparum* Isolates during Erythrocyte Schizogony

**DOI:** 10.1371/journal.pone.0049540

**Published:** 2012-11-15

**Authors:** Anna Bachmann, Michaela Petter, Ann-Kathrin Tilly, Laura Biller, Karin A. Uliczka, Michael F. Duffy, Egbert Tannich, Iris Bruchhaus

**Affiliations:** 1 Department of Molecular Parasitology, Bernhard Nocht Institute for Tropical Medicine, Hamburg, Germany; 2 Department of Medicine, Royal Melbourne Hospital, Parkville, Australia; University of Copenhagen, Denmark

## Abstract

Avoidance of antibody-mediated immune recognition allows parasites to establish chronic infections and enhances opportunities for transmission. The human malaria parasite *Plasmodium falciparum* possesses a number of multi-copy gene families, including *var*, *rif*, *stevor* and *pfmc-2tm,* which encode variant antigens believed to be expressed on the surfaces of infected erythrocytes. However, most studies of these antigens are based on *in vitro* analyses of culture-adapted isolates, most commonly the laboratory strain 3D7, and thus may not be representative of the unique challenges encountered by *P. falciparum* in the human host. To investigate the expression of the *var*, *rif-A*, *rif-B*, *stevor* and *pfmc-2tm* family genes under conditions that mimic more closely the natural course of infection, *ex vivo* clinical *P. falciparum* isolates were analyzed using a novel quantitative real-time PCR approach. Expression patterns in the clinical isolates at various time points during the first intraerythrocytic developmental cycle *in vitro* were compared to those of strain 3D7. In the clinical isolates, in contrast to strain 3D7, there was a peak of expression of the multi-copy gene families *rif-A*, *stevor* and *pfmc-2tm* at the young ring stage, in addition to the already known expression peak in trophozoites. Furthermore, most of the variant surface antigen families were overexpressed in the clinical isolates relative to 3D7, with the exception of the *pfmc-2tm* family, expression of which was higher in 3D7 parasites. Immunofluorescence analyses performed in parallel revealed two stage-dependent localization patterns of RIFIN, STEVOR and *Pf*MC-2TM. Proteins were exported into the infected erythrocyte at the young trophozoite stage, whereas they remained inside the parasite membrane during schizont stage and were subsequently observed in different compartments in the merozoite. These results reveal a complex pattern of expression of *P. falciparum* multi-copy gene families during clinical progression and are suggestive of diverse functional roles of the respective proteins.

## Introduction

During erythrocyte schizogony, the human malaria parasite *Plasmodium falciparum* modifies the host cell surface, and these alterations have been linked to immune evasion and *falciparum* malaria pathology. Morphologically, the most striking change is the emergence of electron dense knobs from the erythrocyte membrane in which parasite-derived variant surface antigens (VSAs) are anchored [1]. VSAs displayed on the surface of infected erythrocytes (IEs) mediate binding to endothelial cells, thereby enabling the parasite to avoid passage through the spleen, recognition and subsequent killing [2], [3], [4]. However, these parasite-encoded membrane proteins are exposed to the host immune system. *P. falciparum* VSAs are encoded by multi-copy gene families, which enables successive expression of individual variants in a process called antigenic variation that allows the parasites to evade the host immune response and establish chronic infection. The high molecular weight *P. falciparum* erythrocyte membrane protein 1 (*Pf*EMP1) family, for example, is encoded by 60 *var* genes per parasite genome and is well known to be involved in immune evasion of IEs. Members of the *Pf*EMP1 family have been shown to mediate adhesion of IEs to several endothelial receptors such as CD36 [5], [6], intercellular adhesion molecule 1 (ICAM-1) [7], vascular cell adhesion molecule 1 (VCAM-1) [8], chondroitin-4-sulfate A (CSA) [9], [10], and P- and E-selectin [8], [11]. The rate and mechanism of antigenic variation of *Pf*EMP1 on IEs is currently an area of active research, but gene structure, regulatory elements, epigenetic memory and subnuclear localization seem to play a role in this process (reviewed in [12]). Much less is known about the other putative VSAs of *P. falciparum*, such as RIFIN, STEVOR and *Pf*MC-2TM. These proteins are members of the two transmembrane (2TM) superfamily based on a shared transmembrane topology that includes a central hypervariable loop sequence [13], [14], [15]. To date, 143 *rif* (repetitive interspersed family), 32 *stevor* (subtelomeric variable open reading frame) and 13 *pfmc-2tm* (*P. falciparum* Maurer’s clefts 2 transmembrane) genes have been identified in the 3D7 reference genome. These genes are expressed during red blood cell schizogony and in other developmental stages of *P. falciparum*, including sexual blood stages, as well as in infective sporozoites, which indicates that these genes are functionally important during most phases of the *P. falciparum* life cycle [15]–[24]. RIFIN proteins can be divided into A- and B-type RIFINs depending on the presence or absence of a semi-conserved 25 amino acid motif. They localize to different compartments of IEs and are predicted to have different functions. A-type RIFIN proteins are associated with the Maurer’s cleft, a membranous network that is involved in the export of proteins from the parasite cytosol to the IE surface. B-type RIFIN proteins, on the other hand, appear to be mostly retained inside the parasite [16], [25], [26]. Members of the STEVOR and *Pf*MC-2TM protein families localize to Maurer’s clefts and to knobs protruding from the erythrocyte membrane [22], [23], [27], [28]. RIFIN and STEVOR proteins are also found in different compartments in merozoites during the human blood phase. Similar to their distinct localization patterns in trophozoites, in merozoites, A-type RIFINs are located at the apical tip, whereas B-type RIFINs are cytosolic [29]. Interestingly, STEVOR proteins can be observed at either the apical tip or the parasite surface during the invasive stage, depending on the antisera and parasite strain used for detection [22], [23], [24].

During erythrocyte schizogony, the expression of multi-copy gene families seems to occur in a highly organized, sequential manner starting with the transcription of *var* genes at the ring stage (3–18 hours post infection, hpi) [30], [31], followed by the expression of *rif* genes (12–27 hpi) [30], [31], [32], [33] and finally transcription of the *pfmc-2tm* (18–30 hpi) [14] and *stevor* (22–32 hpi) [14], [34] gene families. *Pf*EMP1 proteins appear on the surface of IEs as the parasite reaches the trophozoite stage and mediate sequestration at vascular sites, allowing the IE to leave the blood circulation. The role of the 2TM superfamily members in the process of cytoadherence remains unknown, but their expression patterns seem to suggest a later role in the erythrocytic development of the parasite. However, the loss of sequestration phenotype observed in a splenectomized patient was associated with the lack of *var*, *rif-A* and *stevor* expression [26] and, with the exception of *rif*, all genes of the 2TM multi-copy gene families are expressed in a clonally variant manner [14], [35], which suggests that these protein families may be involved in the processes of sequestration and antigenic variation [36], [37], [38]. Nevertheless, the biological functions of the RIFIN, STEVOR and *Pf*MC-2TM proteins during the blood phase of malaria infection are largely unknown, due for the most part to a lack of functional analyses.

Despite improvements in our knowledge over the past few years, little information is currently available on the expression dynamics of VSA-encoding multi-copy gene families in clinical *P. falciparum* isolates. This is an important gap in our understanding of parasitic multi-copy gene families, since *var*, *rif*, *stevor* and *pfmc-2tm* are believed to be involved in immune evasion strategies like antigenic variation and sequestration, neither of which can be effectively simulated *in vitro*. Additionally, IE cytoadherence and concomitant expression of VSAs have been shown to be influenced strongly by adaptation to prolonged cultivation *in vitro* of patient isolates [16], [39], [40]. To overcome this potential bias of *in vitro* studies, we analyzed *ex vivo* samples of clinical isolates from non-immune malaria patients along with samples collected at multiple time points throughout the first *in vitro* generation of the respective isolates. Freshly isolated parasites were compared to the laboratory strain 3D7 by quantitative analysis of VSA expression and analysis of VSA protein localization during the intraerythrocytic developmental cycle. We observed striking differences between the laboratory and clinical strains, including a second *rif-A*, *stevor* and *pfmc-2tm* expression peak in young ring stage parasites in clinical isolates but not in the 3D7 strain, and two distinct patterns of protein expression in young trophozoites and schizonts. These two temporally divergent protein populations were differentially localized, implying that they had divergent functions. Additionally, considerably higher expression levels of *var*, *rif* and *stevor* genes were observed in clinical isolates compared to the laboratory strain, which supports the idea that the respective VSAs are involved in key processes in the natural host environment. In contrast, transcripts of the *pfmc-2tm* gene family were more abundant in the culture-adapted strain, an observation that suggests other, as yet unidentified, functions of the smallest of the multi-copy gene families.

## Results

### Novel Quantitative Real-time PCR Approach to Analyzing the Overall Expression Patterns of Multi-copy Gene Families Encoding VSAs in *P. falciparum* Isolates

**Figure 1:**
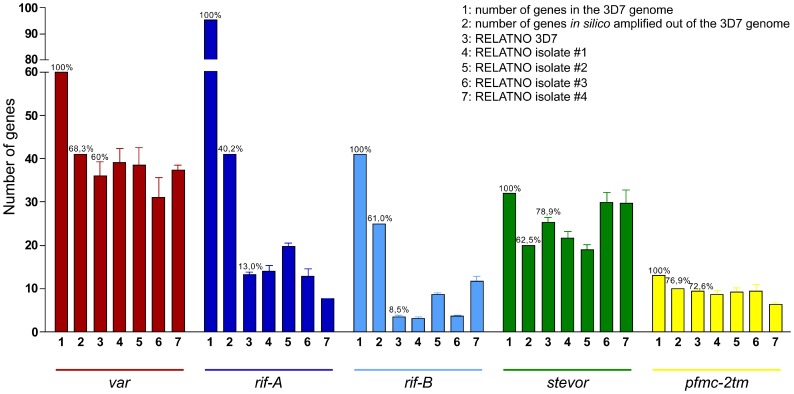
Validation of the degenerate primer pairs used in quantitative real-time PCR. Primer pairs targeting the multi-copy gene families *var*, *rif-A*, *rif-B*, *stevor* and *pfmc-2tm* were designed to amplify a broad repertoire of different genes present in the 3D7 genome (first bar, 1), as indicated by *in silico* PCR results (second bar, 2). Experimental validation revealed similar numbers of genes amplified by the indicated primer pairs in every *P. falciparum* genotype relative to single-copy *fructose-bisphosphate aldolase* (RELATNO) (third to seventh bar, 3–7). *In silico* PCR results were experimentally confirmed for the *var* (red), *stevor* (green) and *pfmc-2tm* (yellow) primer pairs; however, the *rif-A* (dark blue) and *rif-B* (light blue) primer pairs only partially covered the genomic repertoire. Shown are mean values and standard deviations obtained by analysing two biological samples of 3D7 and *ex vivo* isolated gDNA of the clinical isolates in quadruplicates. 1: Number of genes present in the 3D7 genome (set as 100%); 2: number and percentage of genes amplified *in silico* using the one mismatch configuration (http://insilico.ehu.es); 3–7: experimentally calculated RELATNOs of amplified genes of the indicated multi-copy gene families using gDNA from the 3D7 laboratory strain (3) (including percentages) and from clinical isolates #1 (4), #2 (5), #3 (6) and #4 (7).

Quantitative analysis of overall transcription of the multi-copy gene families in different *P. falciparum* clinical isolates was carried out using degenerate primer pairs designed to amplify semi-conserved regions of the *var* [16], [41], *rif-A*, *rif-B*, *stevor* and *pfmc-2tm* genes. *In silico*-simulated PCR using universal primer pairs amplified 68.3%, 40.2%, 61.0%, 62.5%, and 76.9% of the *var*, *rif-A*, *rif-B*, *stevor*, and *pfmc-2tm* gene repertoire in the 3D7 genome, as determined by the one mismatch configuration (http://insilico.ehu.es) (Fig. 1, second bar). These data were experimentally validated using genomic DNA from 3D7 parasites as well as from clinical isolates #1–#4. The number of amplified genes (RELATNO) was calculated relative to *fructose-bisphosphate aldolase*, a single copy gene. Using degenerate primer pairs, 36.0 *var*, 13.2 *rif-A*, 3.5 *rif-B*, 25.2 *stevor*, and 9.4 *pfmc-2tm* genes on average were amplified from 3D7 genomic DNA using the real-time PCR approach. These numbers corresponded to an amplification of 60.0%, 13.0%, 8.5%, 78.9%, and 72.6% of the total gene repertoire of the respective gene family in strain 3D7 (Fig. 1, third bar). Amplification of various *var*, *stevor* and *pfmc-2tm* genes from 3D7 genomic DNA was confirmed by cloning and sequencing of the PCR products (Table S1). Next, PCR was carried out using genomic DNA from the clinical isolates as a template in order to control for primer pair performance in different *P. falciparum* isolates. Nearly the same number of genes was amplified in every genotype and the numbers were similar to those observed for 3D7. On average, 36.4 (±2.9) *var*, 13.5 (±3.8) *rif-A*, 6.1 (±3.5) *rif-B*, 25.1 (±4.3) *stevor* and 8.6 (±1.2) *pfmc-2tm* genes were amplified per *P. falciparum* genotype (Fig. 1, bars 4 to 7).

Thus, using this quantitative real-time PCR approach, we were able to amplify a broad range of genes using degenerate primers targeting different multi-copy gene families in *P. falciparum* isolates with diverse genomic backgrounds. *Rif* subgroups A and B were amplified with lower efficiency than the other multi-copy gene families, and the corresponding primer pairs only partially covered the gene repertoire (Table S2; Fig. 1).

### Transcription of 2tm Superfamily Genes Peaks Twice during Intraerythrocytic Development in Clinical *P. falciparum* Isolates

**Figure 2:**
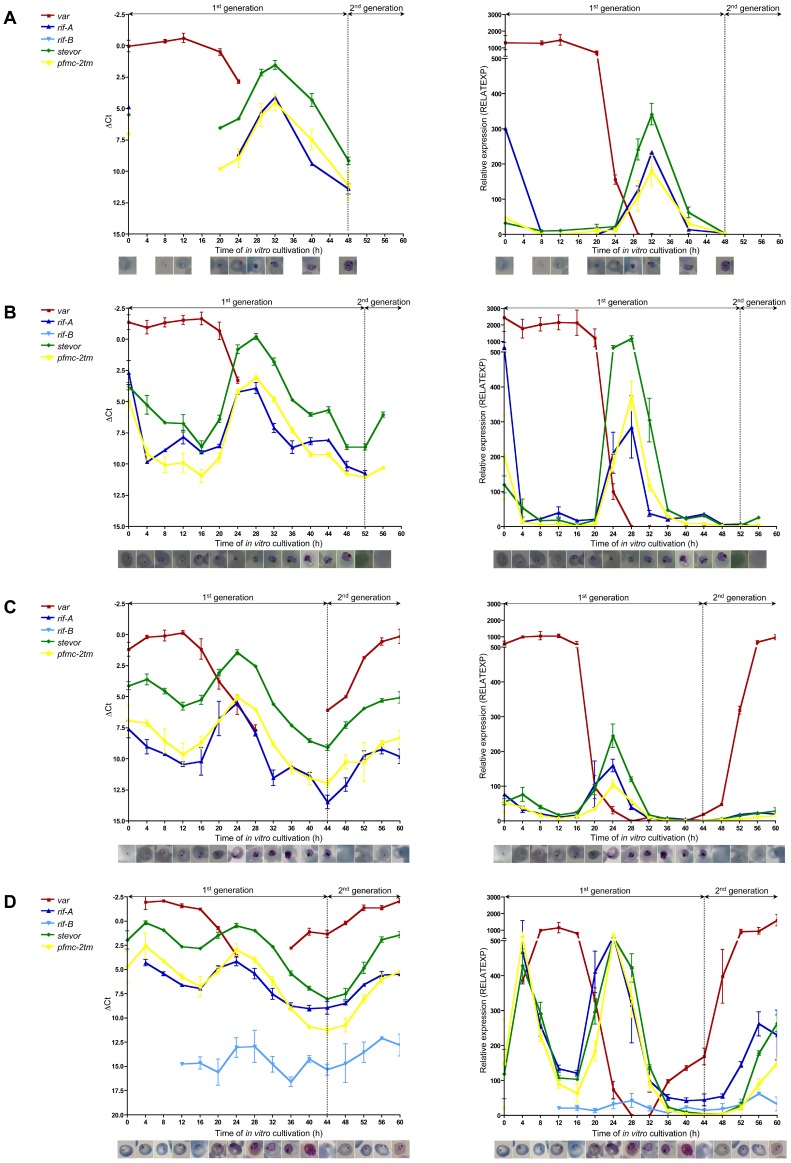
Expression profiles of multi-copy gene families in clinical *P. falciparum* isolates. **A–D:** Expression profiles of the multi-copy gene families *var* (red line, squares), *rif-A* (dark blue line, triangles), *rif-B* (light blue, upside-down triangles), *stevor* (green line, diamonds), and *pfmc-2tm* (yellow line, dots) are represented by either ΔCt values (left chart) or relative expression values (RELATEXP) calibrated against the corresponding gDNA and corrected for amplification efficiency (right chart). Error bars represent the standard deviation of the mean obtained by analysing duplicates of both samples harvested for each time point during erythrocyte schizogony. Two expression peaks were observed for the *rif-A*, *stevor* and *pfmc-2tm* gene families in rings and trophozoites obtained from clinical isolates #1 (**A**), #2 (**B**), #3 (**C**) and #4 (**D**). *Var* gene expression was observed exclusively in ring stage parasites. Expression was normalized to the reference gene *fructose-bisphosphate aldolase* (PF14_0425). Times of *in vitro* cultivation (h) and Giemsa-stained parasites at each time-point are plotted on the x-axis.

To determine the expression of multi-copy gene families during erythrocyte schizogony, quantitative real-time PCR was used to analyze four of the clinical isolates as well as two biological samples of strain 3D7. Samples were collected every 4 h and expression profiles are presented either as ΔCt or relative expression (RELATEXP). The linear range of ΔCt clearly showed all expression fluctuations, while relative expression values, which account for exponential amplification during qPCR as well as the amplification efficiencies of the primer pairs, allowed a comparison of different primer pairs. Both methods demonstrated that *var, rif-A, stevor* and *pfmc-2tm* genes were expressed in the *ex vivo* samples at 0 hours, which represents young ring stage parasites, in all of the clinical isolates investigated. While expression of the *var* gene family was sustained at a high level during the ring stage and up to 20 h in culture, the expression levels of the *rif-A*, *stevor* and *pfmc-2tm* gene families declined rapidly, reaching a minimum at around 12–16 h, before rising to a second peak of transcription after 24 to 32 h of cultivation when all isolates reached the mid-age trophozoite stage (Fig. 2). Interestingly, the expression of all *2tm* superfamily members peaked at the same time in ring stage parasites as well as in trophozoites in every isolate investigated, which indicated that A-type *rif*, *stevor* and *pfmc-2tm* are expressed simultaneously rather than sequentially. To rule out the possibility that the first transcription peak was due to an overall higher expression level of the multi-copy gene families *in vivo* compared to parasites maintained *in vitro*, two isolates (#3, #4) were continuously cultivated until they reached the ring stage of the second *in vitro* generation (Fig. 2). Ring stage parasites of the second generation also showed an increase in specific transcripts, which verified the expression peaks of *rif-A*, *stevor* and *pfmc-2tm* families at this stage in fresh clinical isolates. Furthermore, there was a slight rise in expression of *rif-A* family genes at the schizont stage in isolates #2 and #3 (Fig. 2). Remarkably, no transcripts of the *rif-B* gene family were detected in three of the clinical isolates (#1, #2, #3) at any point in the asexual replication cycle, and *rif-B* expression levels in isolate #4 were the lowest of all the multi-copy gene families analyzed (Fig. 2). This may have been due to the limited coverage of *rif-B* sequences by the primer pair. Nevertheless, a slight increase of *rif-B* transcription was observed in ring stage, mid-age trophozoites and schizonts in isolate #4.

**Figure 3:**
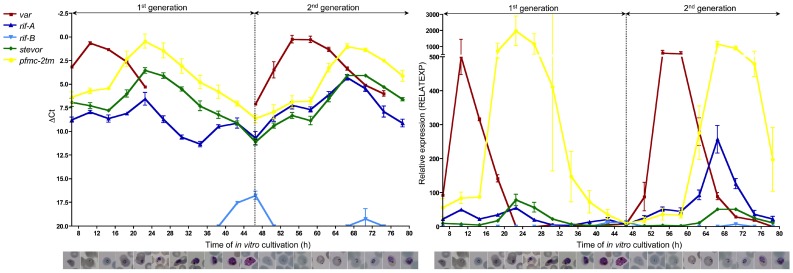
Expression of multi-copy gene families in the 3D7 laboratory strain during erythrocyte schizogony. Expression profiles of the multi-copy gene families *var* (red line, squares), *rif-A* (dark blue line, triangles), *rif-B* (light blue, upside-down triangles), *stevor* (green line, diamonds), and *pfmc-2tm* (yellow line, dots) in long-term *in vitro* cultivated strain 3D7 during erythrocyte schizogony. In contrast to the clinical isolates, 3D7 parasites exhibited a slight increase of *rif-A*, *stevor* and *pfmc-2tm* expression during the ring stage, and the main peak of expression of the multi-copy gene families was observed in mid-age trophozoites. Transcription of the *var* gene family was restricted to ring stage parasites. Expression was normalized to the reference gene *fructose-bisphosphate aldolase* and is represented by either ΔCt (left chart) or relative expression (RELATEXP, right chart). 3D7 time course experiments were performed twice and two samples were analysed for each time point at least in duplicates. Graphically shown are mean values and standard deviations of all qPCR runs performed for the respective time points Parasite developmental age (hpi) and the corresponding parasitic stage (shown as Giemsa staining) are plotted on the x-axis.

To validate these novel findings and compare them to the 3D7 reference strain, which has been used extensively for transcriptome studies, a similar time course experiment was performed twice. 3D7 parasites exhibited a similar expression profile during erythrocyte schizogony as the clinical isolates, with *var* gene expression occurring exclusively in ring stage parasites and two maxima of expression for the *rif-A*, *stevor* and *pfmc-2tm* gene families in ring and trophozoite stage parasites (Fig. 3). The expression peak of the *2tm* multi-copy gene families in the ring stages (10.5 h and 54.5 h) was rather small compared to the peak detected in pigmented trophozoites (22.5 and 66.5 h). Additionally, a third expression peak of *rif-A* gene transcription in schizonts was detected (42.5 h) in 3D7 parasites (Fig. 3). The *rif-B* expression profile observed in clinical isolate #4, marked by increased *rif-B* transcript abundance in rings, trophozoites and schizonts, was also seen in both biological samples of strain 3D7 (Fig. 2 and 3).

### Expression Levels Differ between Laboratory Strain 3D7 and ex vivo Clinical Isolates

**Figure 4:**
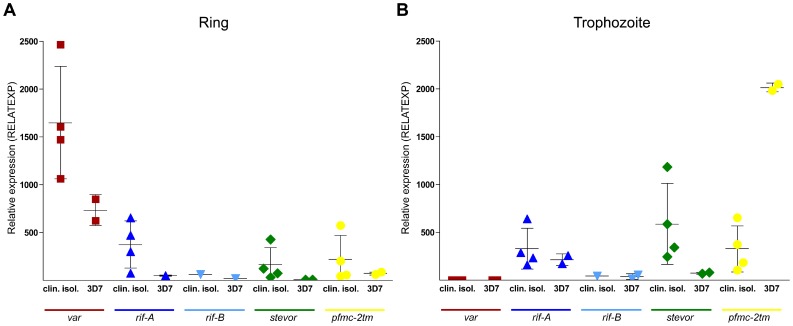
Relative expression levels of multi-copy gene families in clinical isolates compared to the 3D7 strain. **A, B:** Maximum relative expression values of the multi-copy gene families *var*, *rif-A*, *rif-B*, *stevor* and *pfmc-2tm* detected in rings (**A**) and trophozoites (**B**) from clinical isolates #1–4 were plotted against the corresponding values from two biological samples of strain 3D7. Each symbol represents the mean value for each isolate or biological sample. Additionally, the means and standard deviations were shown for the group of clinical isolates and 3D7 samples.

To compare mRNA levels between different isolates, the highest relative expression values in rings and trophozoites of the clinical *P. falciparum* isolates were compared with 3D7 parasites. There was substantial higher expression of *var* (1.5–3.4 fold), *rif-A* (1.5–12.8 fold), *stevor* (4.8–62.5 fold) and *pfmc-2tm* families (0.6–7.8 fold) in the patient isolates relative to 3D7 in ring stage parasites (Fig. 4A). The same trend was also evident for *rif-A* (0.8–2.6 fold) and *stevor* (2.5–16.4 fold) transcripts in the trophozoite stage (Fig. 4B). In contrast, there was an increase (3.1–19.1 fold) in *pfmc-2tm* transcription in 3D7 trophozoites compared to the clinical isolates. No differences were observed between *rif-B* expression levels in rings or trophozoites in clinical isolate #4 and strain 3D7; however, one must keep in mind that *rif-B* family transcripts were not detected in three of the four isolates analyzed (#1, #2, #3) (Fig. 4).

**Figure 5:**
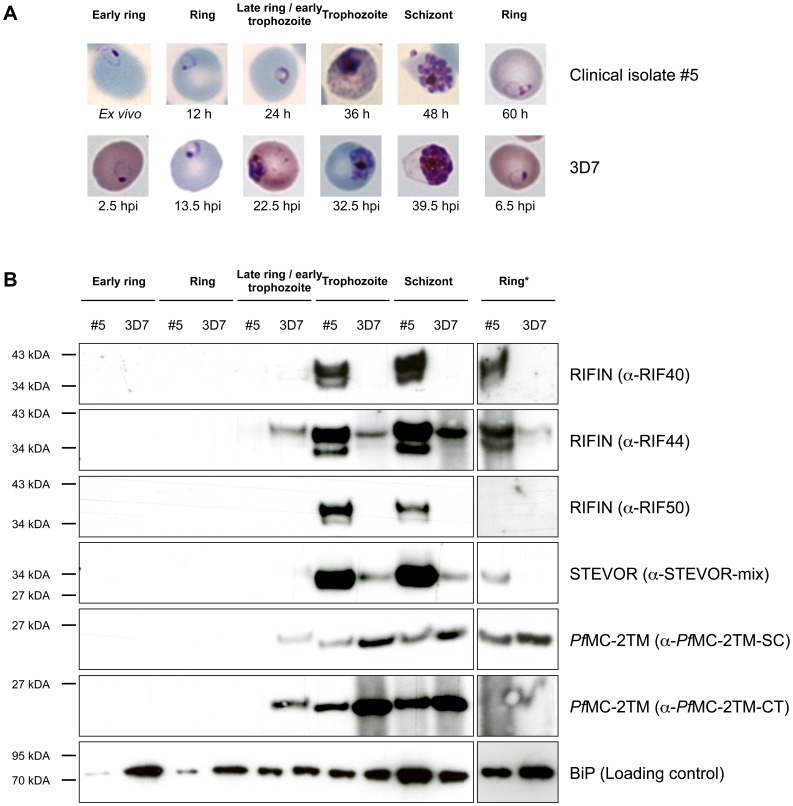
Immunoblot analysis of VSA abundance in the clinical isolate #5 and the 3D7 strain. **A, B**: Isolate #5 (h: time of *in vitro* cultivation) and 3D7 (hpi: hours post infection) at successive developmental stages were harvested (**A**), and differences in VSA abundance in the membrane fraction were assessed by immunoblot using α-RIF40, α-RIF44, α-RIF50, α-STEVOR-mix, α-*Pf*MC-2TM-SC, and α-*Pf*MC-2TM-CT antisera (**B**). As expected, RIFIN and STEVOR were present at higher levels in the clinical isolate; in contrast, *Pf*MC-2TM proteins were quantitatively increased in 3D7 parasites. Differences were most obvious in pigmented parasite stages (trophozoites, schizonts, left), which also exhibited the highest levels of protein during intraerythrocyic development, but upon longer exposure (*), similar results were also observed for ring stage parasites (right). The luminal endoplasmic reticulum (ER) protein BiP (HSP70) served as a loading control.

To validate the results of RNA analysis, saponin lysates from isolate #5 were prepared every 12 h during the first parasite replication cycle *in vitro* and from comparable stages of highly synchronized 3D7 parasites, and analyzed by immunoblot. In the second half of erythrocyte schizogony, RIFIN and STEVOR protein levels were clearly higher in clinical isolate #5 compared to 3D7 parasites when using α-RIF40, α-RIF44, and α-RIF50 antisera as well as αSTEVOR-mix. In contrast, higher levels of *Pf*MC-2TM were detected in laboratory strain 3D7 using two α-*Pf*MC-2TM antisera (Fig. 5). Upon longer exposure, similar results to the mature parasites were obtained for ring stage parasites of clinical isolate 5 compared to 3D7 (Fig. 5). The only time point at which 3D7 parasites seemed to have higher levels of all VSAs was during the transition from late ring stage parasites to trophozoites. However, Giemsa-stained smears of the clinical isolate and strain 3D7 revealed slight differences in parasite development at this particular point: 3D7 parasites had already incorporated hemozoin, while late rings without hemozoin were observed in smears of clinical isolate #5 (Fig. 5A). Taken together, these results demonstrated that the differences in RNA abundance between the clinical isolates and 3D7 correlated with differences in protein expression profiles.

### Expression of the Same Gene Variants in Ring and Trophozoite Stage Parasites

To investigate whether the two expression peaks we observed were due to the transcription of different subsets of genes or the same genes, cDNA from the clinical isolates and from both biological samples of 3D7 was isolated during the peaks of expression and then subjected to amplification using universal primers spanning longer and more variable regions than the primers used for real-time PCR analysis [26]. To control for potential primer bias towards preferentially amplified genes, gDNA was analyzed in parallel (Fig. S2; Table S3). Amplicons were cloned and 15 bacterial colonies on average were sequenced to determine the identity of the expressed genes. Statistical analysis revealed no apparent differences in the array of genes expressed in ring stage and trophozoites, although in isolate #3, there was a switch in the dominant *pfmc-2tm* variant expressed from sequence #1 in the *ex vivo* ring stage to sequence #6 in the trophozoite stage and subsequent ring stage of the second generation (Fig. S2). However, in this particular case, a primer bias towards sequence #6 was observed at the genomic level, so any potential switching event should be carefully considered in this context (Table S3).

### Discrete Subcellular Localization of VSAs during Erythrocyte Schizogony

**Figure 6:**
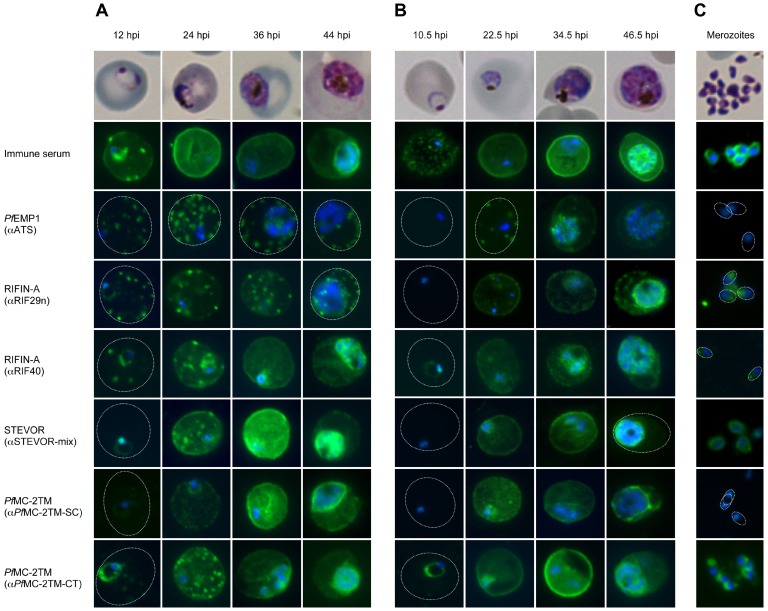
Localization of VSAs during the intraerythrocytic developmental cycle. **A-C:** Representative immunofluorescence images of the indicated VSAs in different parasite developmental stages of clinical isolate #4 (**A**), 3D7 parasites (**B**), and free merozoites from isolate #1 (**C**). First row: Giemsa staining of the corresponding parasitic stage. Second row: Positive control serum obtained from a semi-immune patient. Third row: *Pf*EMP1-specific antibody, showing the presence of the protein in Maurer’s clefts over the entire time course (**A, B**). Third to eighth rows: 2TM proteins were exported into the host cell (12–36 hpi) during the trophozoite stage but remained inside the parasite in the schizont stage (48 hpi). Proteins of the RIFIN-A family frequently localized to Maurer’s clefts, particularly when using the α-RIF29n antiserum, and the erythrocyte membrane; STEVOR and *Pf*MC-2TM localized predominantly to the erythrocyte membrane (**A, B**). RIFIN and STEVOR proteins were also observed at the apical tip or at the merozoite membrane, respectively. Isolate #1 also exhibited *Pf*MC-2TM-specific fluorescence in free merozoites when using the α-P*f*MC-2TM-CT antiserum (**C**). All antibodies were visualized with Alexa488-conjugated secondary antibody (green), and nuclei were stained with DAPI (blue).

In parallel to RNA sampling, smears were prepared for indirect IFA to monitor protein expression and localization at different time points of parasite development. VSA localization was quantified to obtain a more accurate assessment, and immune serum from a semi-immune malaria patient was used as a positive control to monitor the location of immunogenic proteins during erythrocyte schizogony. These proteins were exported early in parasite development, as evidenced by strong fluorescence signals at the erythrocyte membrane. Additionally, merozoites exhibited positive staining on the surrounding membrane that was already visible during schizont stage, as seen by the accumulation of fluorescence inside the parasite membrane (Fig. 6; Fig. S3). Where possible, multiple antibodies directed against different variants were used for each VSA family (Fig. S3, S4, S5, S6, S7, S8 and S9). We also took advantage of the cross-reactivity of each antiserum with other members of the same protein family, a common phenomenon that is useful in particular for detection in clinical isolates [42], [43], [44] (Fig. S1). *Pf*EMP1 proteins localized predominantly at the Maurer’s clefts in ring stage parasites through schizont stage, whereas the erythrocyte membrane showed only marginal, if any, staining (Fig. 6A and 6B; Fig. S4). However, several multiple-infected IEs exhibited substantial accumulation of *Pf*EMP1 at the erythrocyte membrane due to the high levels of protein in these cells (data not shown). Interestingly, IFA revealed two populations of A-type RIFIN, STEVOR and *Pf*MC-2TM with distinct localization patterns in IEs. In the trophozoite stage, RIFIN proteins were exported predominantly to the Maurer’s clefts and the erythrocyte membrane, whereas STEVOR and *Pf*MC-2TM proteins seemed to localize predominantly to the IE membrane (Fig. 6A, 6B; Figs. S5, S6, S7 S8, and S9). In schizonts, the fluorescence intensity of the exported protein fraction became less intense and protein accumulated within the parasite boundaries. A-type RIFINs, STEVORs and *Pf*MC-2TMs were located in close proximity to the nuclei in dividing parasites in the clinical isolates. In strain 3D7 schizonts, *Pf*MC-2TM family proteins exhibited two distinct localization patterns depending on the antisera used for detection. *Pf*MC-2TM proteins appeared to associate with the parasitophorous vacuole membrane or parasite membrane when using α-*Pf*MC-2TM-SC, while staining with α-*Pf*MC-2TM-CT resulted in a fluorescence pattern similar to RIFIN and STEVOR (Fig. 6A, 6B; Figs. S5, S6, S7, S8, and S9). Using α-*Pf*MC-2TM-CT serum, *Pf*MC-2TM proteins were detected only in free merozoites from isolates #1 and #3, whereas A-type RIFIN and STEVOR proteins were detected in released merozoites from all *P. falciparum* isolates examined, either at the apical tip harbouring the organelles necessary for host cell invasion or the surrounding merozoite membrane, respectively (Fig. 6C). The association of A-type RIFIN and STEVOR proteins with merozoites was substantiated by immunoblot analysis (Fig. S10), whereas the presence of *Pf*MC-2TM proteins could not be confirmed in merozoites isolated from 3D7 or four of the clinical isolates (#1, #3, #4 and #5), even after several rounds of parasite replication *in vitro* (data not shown). The localization of VSAs was confirmed by co-staining with antisera specific for markers of subcellular compartments in IEs and free merozoites (Fig. S11).

Among the isolates, there were slight differences in protein staining patterns. For example, VSAs localized more frequently at Maurer’s clefts in isolate #1, which also exhibited the most prominent VSA-positive staining in merozoites. The most striking discrepancy among the clinical isolates and 3D7 was the increased number of cells with exported *Pf*MC-2TM proteins; in 3D7 parasites at 34.5 hpi, on average, 87.9% of *Pf*MC-2TM proteins was exported beyond the parasitophorous vacuole membrane compared to 18.3% in 36 h-cultures of clinical isolates, in which *Pf*MC-2TM proteins localized to the Mauer’s clefts or erythrocyte membrane, regardless of which *Pf*MC-2TM antiserum was used (Table 1). Furthermore, parasites freshly isolated from patients exhibited a high proportion of *Pf*EMP1- and A-type RIFIN-positive ring stage parasites and trophozoites, whereas positive staining for exported VSAs was less frequent and intense in 3D7 parasites at comparable stages. STEVOR staining was very bright in all isolates and in strain 3D7, but quantification of the IFA data suggested a slight increase in exported protein in the clinical isolates at three of the four time points examined (Table 1) (Figs. S4, S5, S6, S7, S8, and S9). Furthermore, the clinical isolates exhibited a higher proportion of *Pf*EMP1, RIFIN and STEVOR proteins at the Maurer’s clefts and erythrocyte membrane during schizont stage compared to strain 3D7 (Table 1) (Figs. S4, S5, S6, and S7).

**Table 1:** Proportion of infected erythrocytes with VSAs exported to host cell compartments in clinical isolates and 3D7 at different developmental stages.

	Clinical isolates	3D7	Clinical isolates	3D7	Clinical isolates^1^	3D7	Clinical isolates^2^	3D7
	12 h	10.5 hpi	24 h	22.5 hpi	36 h	34.5 hpi	48 h	46.5 hpi
***Pf*** **EMP1**	67.5%	5.8%	69.4%	19.0%	81.1%	71.7%	92.3%	84.5%
**RIFIN-A**	50.6%	1.0%	68.2%	36.7%	73.7%	62.9%	74.0%	38.0%
**STEVOR**	5.3%	0%	47.9%	34.7%	79.4%	91.1%	81.0%	38.2%
***Pf*** **MC-2TM**	3.0%	0%	7.6%	43.9%	18.3%	87.9%	20.0%	68.9%

1 Time point not determined for isolate #1.

2 Time point of 44 h for isolate #4.

hpi, hours post-infection.

## Discussion

Successful *in vitro* culture of *P. falciparum* is a cornerstone of modern malaria research and has been instrumental in increasing our knowledge about this life-threatening disease [45]. Nevertheless, *in vitro* culture systems do not fully recapitulate all of the important interactions between the parasite and its human host, and, to date, an appropriate animal model for *P. falciparum* is not available. The *Pf*EMP1, RIFIN, STEVOR and *Pf*MC-2TM protein families are unique to *P. falciparum* and it’s nearest relative, *P. reichenowi*; thus, it is not clinically or biologically feasible to use other *Plasmodium* species to unravel the biological functions of these proteins *in vivo*. The immune system can play an important role in orchestrating the sequential display of VSAs once an infection is established. Genes encoding the VSAs are characterized by a high degree of genomic variability and are highly polymorphic between strains and even within a single strain [13], [46]. Bozdech *et al.* performed a microarray-based comparative genomic hybridization analysis using gDNA from strains 3D7 and HB3 to determine regions of genomic variability. They demonstrated that the chromosomal regions containing VSA-encoding genes are regions with the highest degree of genetic variation. Only 28.3% of the *rif* gene repertoire, 41.7% of the *var* gene repertoire and 51% of the *stevor* gene repertoire of 3D7 were recognized by HB3 gDNA by spot array hybridization, limiting the type of analysis [47]. Consequently, multi-copy gene families have usually been excluded from transcriptional analyses of isolates other than 3D7 or are handled separately [30], [47]–[50]. We have developed a quantitative real-time PCR technique that uses degenerate primer pairs targeting conserved regions within the *var*, *rif-A*, *rif-B*, *stevor*, and *pfmc-2tm* multi-copy gene families to investigate these highly variable genes in clinical isolates. Using this approach, we were able to analyze and compare the timing and level of expression of the various groups of VSA genes during the entire erythrocytic replication cycle of clinical isolates of *P. falciparum* with different genomic backgrounds.

Applying this approach to freshly isolated *P. falciparum* isolates as well as the 3D7 laboratory strain, we observed striking differences in VSA expression profiles throughout the intraerythrocytic developmental cycle. Chief among them was the higher expression of genes encoding surface proteins such as *Pf*EMP1, STEVOR and A-type RIFIN in patient isolates compared to 3D7. Previous work has shown that surface protein expression is higher in *P. falciparum* field isolates compared to culture-adapted strains like 3D7 [16], [22], [37], [48], [50]. Here, we showed that 3D7 trophozoites had higher transcription levels of *pfmc-2tm* family genes compared to clinical isolates, suggesting divergent functions for *Pf*MC-2TM proteins. The quantitative RNA data were confirmed by immunoblot analysis on protein level. Second, based on RNA analysis, we observed an additional expression peak of the *2tm* superfamily genes *rif-A*, *stevor* and *pfmc-2tm* in young ring stage parasites in clinical isolates, whereas the 3D7 laboratory strain displayed only a small expression plateau of these multi-copy gene families in the ring stage. In line with this observation, all VSAs were over-expressed in clinical isolates during the ring stage. Apart from this previously undetected second transcription peak in the ring stage, the timing of expression of the *var*, *rif*, *stevor* and *pfmc-2tm* families was consistent with the literature, with *var* gene expression occurring in ring stage parasites and the *2tm* transcripts peaking in trophozoites [27], [30], [31], [32], [33], [34]. However, in contrast to what has previously been suggested, we showed that the expression of the multi-copy gene families was not a cascade-like progression in which transcription of *var* genes is followed by *rif*, *stevor*, and finally *pfmc-2tm* expression [51], [52]. Rather, we showed that *var* transcription was for the most part restricted to ring stage parasites, while the expression of the *2tm* superfamily genes occurred synchronously in young ring stage as well as in mid-age trophozoites. Recently, the maximum peak of *var* gene transcription was shown to vary among field isolates, but the majority of isolates had higher expression levels in 20 h cultivated parasites (trophozoites, 22–26 hpi) compared to *ex vivo* parasites (ring stage, 4–10 hpi) [53]. We were not able to corroborate this previous result, and in fact, we demonstrated that *var* gene expression remains consistently elevated during ring differentiation, becoming drastically reduced in every isolate when parasites reached the trophozoite stage. Expression of the *2tm*-superfamily in trophozoites seemed to vary among the isolates with respect to time of *in vitro* culture (24–32 h). However, the results of Giemsa staining suggested that these differences are most likely due to different cycle lengths of the isolates during *in vitro* adaptation (44–52 h) and that this may be an intrinsic property of the isolates.

VSA localization was investigated by indirect IFA to determine whether protein expression correlated with the transcription peaks of the *var*, *rif*, *stevor* and *pfmc-2tm* genes. *Pf*EMP1 proteins were observed at Maurer’s clefts in transit through the erythrocyte cytoplasm early in erythrocyte development, supporting their role in trophozoite sequestration. The proportion of *Pf*EMP1-positive young trophozoites was markedly increased in the clinical isolates compared to the 3D7 laboratory strain, in agreement with the RNA data. The clinical isolates, as well as 3D7 parasites exhibited only marginal accumulation of protein at the IE membrane, as already observed [54], [55], [56]. A more complex picture emerged for the 2TM protein families. In the clinical isolates, we observed two stage-dependent populations of A-type RIFIN, STEVOR and *Pf*MC-2TM proteins, which were either exported into the host cell or retained inside the parasite boundary. A recent study by Foth *et al.* showed that the abundance of most parasite proteins peaks significantly later than the corresponding transcripts, with a median delay of 11 h [57]. Based on this observation, proteins derived from the first peak of RNA in ring stage parasites would be detectable in trophozoites, whereas proteins derived from transcripts generated in the trophozoite stage would be detectable in the early schizont stage of the parasite. In fact, this is what we observed for the 2TM protein families. Additionally, both of the time-delayed protein populations showed distinct patterns of localization, with the first wave of RIFIN, STEVOR and *Pf*MC-2TM proteins localizing to the Maurer’s clefts and the erythrocyte membrane [15], [22], [27], [28], [29], [32], [58], [59], and the second wave exhibiting strong intracellular parasite signals [22], [23], [24], [29]. Subsequently, RIFIN and STEVOR proteins were detected in free merozoites, which can occasionally be found in smears of late stage parasites. RIFIN proteins were observed at the apical tip harbouring the invasion-related organelles, as previously described [29], whereas positive α-STEVOR-mix staining was detected at the merozoite membrane. In fact, STEVOR proteins can be detected outside of IEs in pigmented parasite stages [28] and were recently detected on the merozoite surface [24]. Using an antiserum (α-PFL2610w) that was one component of the α-STEVOR-mix used in the current study, Khattab *et al* was able to detect multiple STEVOR variants throughout the intraerythrocytic developmental cycle, and two protein bands of 29.8 and 31.7 kDa were visible in rings, trophozoites and merozoites [24]. These results suggest that some STEVOR variants are expressed throughout the entire replication cycle and argue against differential regulation of expression of protein variants in different parasitic stages. In agreement with this, most of sequences identified during the two expression peaks in ring and trophozoite stages were identical in the clinical isolates resulting in similar expression patterns. Although 3D7 appeared to express different gene variants in the ring and trophozoite stages, differences in sequence frequency were not statistically significant in either of the two biological samples. Moreover, the two 3D7 biological samples expressed different genes, most likely due to the random expression of many VSA-encoding genes during prolonged *in vitro* cultivation [16], [39], [40]. Based on the data obtained from the clinical isolates, we hypothesize that protein localization is highly dependent on the time point/stage of expression, as previously suggested for RESA and AMA-1 proteins [60], [61], [62]. Accordingly, a single protein variant can be exported into the erythrocyte in trophozoites and subsequently be retained in the parasite for insertion into merozoite membranes during the schizont stage depending on the time point of RNA expression in ring stage parasites and trophozoites. This would be similar to the trafficking of *Pf*EMP1 to the IE surface, which is arrested in mature parasites, resulting in a significant proportion of *Pf*EMP1 in an intracellular pool [54]. Thus, there may be stage-dependent events that limit *Pf*EMP1 export. Alternatively, proteins exported to erythrocytic membrane compartments in trophozoites could be transported in a retrograde fashion back into the parasite at the schizont stage. Additional studies are needed to determine which mechanism may be at play, perhaps involving *P. falciparum* transfectants expressing *2tm* gene variants under the control of different stage-specific promoters or pulse-chase experiments.

Any functional implications of the current results would be highly speculative but the data provide some evidence that A-type RIFIN and STEVOR proteins may be involved in immune evasion strategies, similar to *Pf*EMP1. First, expression of the *rif-A* and *stevor* multigene families was increased in clinical isolates, similar to *var* genes. Genes that are over-expressed *in vivo* may be required for parasite survival in an environment rich in immune cells and other adverse factors, as opposed to the optimized growth conditions seen *in vitro*. Second, A-type RIFIN and STEVOR proteins localized to the erythrocyte membrane, a phenotype that was highly conspicuous in mid-age trophozoites. Thus, these proteins were expressed at the right time and in the right place for a role in immune evasion mechanisms such as antigenic variation or sequestration of IEs at vascular sites. In line with the latter, the recently-modelled protein structure of the RIFIN proteins indicates that the A-type proteins contain an “Armadillo-like” fold, which is known to promote protein-protein interactions [63].

In contrast to A-type RIFIN and STEVOR proteins, the expression of *Pf*MC-2TM proteins was lower in the clinical isolates compared to strain 3D7, although the proteins localized to the erythrocyte membrane. However, several attempts to demonstrate the surface exposure of *Pf*MC-2TM proteins have been unsuccessful, perhaps due to its orientation in the lipid bilayer with only the small hypervariable loop located outside of the membrane [27], [64]. Localization of *Pf*MC-2TM proteins at the erythrocyte membrane was observed more frequently in 3D7 parasites compared to clinical isolates, although we cannot rule out differences in the specificity of the antisera as a reason for this discrepancy. In a previous study, promoter titration failed to completely abolish *pfmc-2tm* expression, whereas it was effective in inhibiting expression of the *var*, *rif* and *stevor* families. The authors suggested that the inability to drive down endogenous *pfmc-2tm* expression may reflect a requirement for this protein family in parasite viability [51]. In another line of evidence, a *P. falciparum* strain isolated from a splenectomized patient lacked *var*, *rif-A* and *stevor* transcripts and had a non-sequestration phenotype *in vivo* and *in vitro*. Expression of *pfmc-2tm* genes was detected in the isolate, indicating either a failure of the immune system to recognize the small region of *Pf*MC-2TM exposed on the erythrocyte surface, or an essential role for *Pf*MC-2TM in parasite survival. Interestingly, members of this protein family have been implicated as transporters, channels or receptors due to the presence of a conserved proline residue in at least one of their transmembrane domains [15]. 2TM superfamily proteins have also been implicated in the formation of the parasite-induced new permeation pathway (NPP); in particular, the positively charged cytoplasmic domain of these proteins [65]. According to currently formulated criteria, proteins involved in the formation of the NPP are highly dependent on the presence of at least two transmembrane helices. This structural feature, while present in members of the *Pf*MC-2TM family, has recently been called into question for the A-type RIFIN and STEVOR proteins [17], [28], [63]. Based on more advanced predictors of secondary structural elements, A-type RIFINs have only one transmembrane helix, and in STEVOR proteins, the semi-conserved region is surface exposed, which is inconsistent with the proposed two transmembrane topology that exposes the variable region on the erythrocyte surface [17], [28], [63].

All 2TM protein families exhibited two peaks of RNA and protein expression, and the two protein populations localized to different sites in the IE. These results suggest a dual role for the corresponding proteins during asexual replication in the human host, with the 2TM proteins perhaps having additional functions in other parasitic stages such as gametocytes and sporozoites [18], [19], [20], [21]. Thus, RIFIN and STEVOR proteins could contribute to immune evasion strategies in IEs and invasive merozoite, with additional roles in host cell recognition, attachment or invasion. *Pf*MC-2TM may participate in nutrient uptake of the parasite through the erythrocyte membrane and in late stages through the PVM [64].

In summary, we demonstrated clear differences between clinical *P. falciparum* isolates and the culture-adapted 3D7 strain in VSA expression patterns. These results underscore the importance of studying host-pathogen interactions in fresh clinical isolates rather than in laboratory strains cultivated *in vitro* in the absence of any immune pressure.

## Materials and Methods

### Ethics Statement

Blood donors providing *P. falciparum* infected erythrocytes were recruited from the diagnostic unit of the Bernhard Nocht Institute for Tropical Medicine, Hamburg, Germany. All patients had given their written informed consent for this study, which was approved by the Ethical Review Board of the Medical Association of Hamburg (reference number PV3828).

Animal use for antisera generation was carried out in strict accordance with the recommendations of the European Union guidelines for the handling of laboratory animals (http://ec.europa.eu/environment/chemicals/lab_animals/home_en.htm). The procedures were approved by the Behörde für Gesundheit und Verbraucherschutz der Stadt Hamburg according to §10a TierSchG (German Protection of Animals Act).

### Parasite Strains and in vitro Culture

Blood samples from *P. falciparum* malaria patients obtained from the diagnostic unit of the Bernhard Nocht Institute for Tropical Medicine, Hamburg, Germany, were used in this study. Gender, age and national residence of the patients providing the clinical isolates as well as the geographical origins and MSP1 genotypes of the isolates are presented in the supporting files (Table S4). Based on native country and national residency, isolates #1 and #4 presumably originated from semi-immune adults, whereas isolates #2, #3 and #5 were isolated from non-immune travellers. Parasitemia of the blood samples ranged from 1% to 3%, with the exception of isolate #5, which contained 36% IEs. Blood (3 to 30 ml) was obtained from the patients and erythrocytes were isolated by ficoll gradient centrifugation. Two aliquots of 400 µl each of red blood cells were separated and further processed for RNA analysis (*ex vivo* samples). The remainder were subsequently cultivated without the addition of allogenic erythrocytes using a protocol adopted from Trager and Jensen [45]. IEs from two culture dishes were harvested for RNA isolation and immunofluorescence analysis (IFA) every 4 h during the first *in vitro* cycle of the isolates.

To obtain tightly synchronized 3D7 parasites, schizonts were separated by magnetic activated cell sorting (MACS). Uninfected erythrocytes were added to allow reinvasion during subsequent cultivation. After 5 h, the culture was synchronized with 5% sorbitol, which allowed only ring stage parasites to survive [66]. The resultant ring stage parasites (2.5 hpi±2.5 hpi) were cultivated in 0^+^ blood as described above. Samples were taken for RNA isolation and IFA, as described for the clinical isolates. The 3D7 time course experiment was repeated twice at an interval of eight months to obtain two independently-cultivated biological samples for the quantitative real-time PCR experiments.

Cultivation and synchronization of isolate #5 and 3D7 for immunoblot analysis was performed as described above, except that sampling intervals were every 12 h for the clinical isolate. Matching 3D7 parasite stages were identified by Giemsa staining and were harvested at 2.5 hpi, 13.5 hpi, 22.5 hpi, 32.5 hpi, 39.5 hpi, and 48.5 hpi. The last sample of both strains (60 h for isolate #5 and 48.5 hpi for 3D7) consisted of ring stage parasites of the next replication cycle.

### RNA Preparation and cDNA Synthesis

Harvested erythrocytes (400 µl) were rapidly lysed in 20 volumes of pre-warmed TRIzol (Invitrogen) and stored at −70°C. RNA was isolated according to the manufacturer’s instructions using the TRIzol Plus RNA Isolation Kit (Invitrogen); DNase treatment was performed using RQ1 RNase-Free DNase (Promega). To confirm the absence of genomic DNA, real-time PCR analysis was carried out using primers specific for cellular *fructose-bisphosphate aldolase* (PF14_0425) [67] and 50 ng of RNA as a template. First strand cDNA synthesis was performed for 2 h at 42°C using random hexamers and the SuperScript III Kit (Invitrogen).

### Quantitative Real-time PCR

Degenerate primer pairs were designed to amplify the entire repertoire of genes from each multi-copy gene family, as follows:

*rif-A*_for: 5′-CCW SAA ATG AAA GAA GTD ATG-3′, *rif-A*_rev: 5′-CTT TRT CRC ATT KWT CTT TAC A-3′; *rif-B*_for: 5′-CGA CAA RCN TCA CAA MGW TT-3′, *rif-B*_rev: 5′-CAC CTC CTA RCC CAC ACC CAC ACH TAA G-3′; *stevor*_for: 5′-CCR CAT TAT CAT AAT GAY CC-3′, *stevor*_rev: 5′-CTA CTA CWT CTT TCA ATT GTT YAT ATG G-3′; and *pfmc-2tm*_for: 5′-GGA ACA TAC AAA YYA TCA TAC CAT AAT-3′, *pfmc-2tm*_rev: 5′-CAA TAT ATT CKT TAA GGY ATT TCC-3′. Expression of *var* was detected using a previously published primer pair [16], [41], and the expression of *fructose-bisphosphate aldolase* was analyzed for normalization [67]. Virtual PCR experiments were performed at http://insilico.ehu.es. The efficiencies (E) of the primer pairs were determined by template dilution of gDNA from 3D7 and three clinical isolates (#1, #3, #4) (Table S2). Efficiencies were validated at the cDNA level using the DART algorithm, as provided on the CAmpER homepage (www.camper.cebitec.uni-bielefeld.de), for each reaction set-up and the mean efficiency for every primer pair was calculated [68]. The efficiency value can be considered an intrinsic property of each primer pair as similar values were obtained by both methods for every isolate. Thus, mean efficiency as determined by template dilution, was used for the correction of relative expression levels (Table S2).

Quantitative amplification was conducted in a Rotor-Gene 3000 (Corbett) using the RealMasterMix SYBR Green Kit (5Prime). Template (50 ng) was mixed with 2.5×RealMasterMix and 0.5 µM sense- and antisense-primer in a final volume of 20 µl. Reactions were incubated at 95°C for 3 min and then subjected to 35 cycles of 95°C for 15 s, 49°C for 20 s and 68°C for 20 s, and a subsequent melting step (67–95°C). The specificity of each primer pair was determined after each PCR run by dissociation curve analysis, and amplification products with evidence of primer dimer formation were excluded from the analysis. The number of amplified genes (RELATNO) for each parasite genotype relative to *fructose-bisphosphate aldolase* (a single-copy gene) was determined using the following formula, which takes into account differences in amplification efficiency:
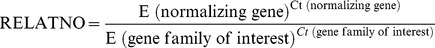



For the time course experiments, data were presented either as ΔCt values (Ct_gene family of interest_−Ct_normalizing gene_) or were corrected for amplification efficiency of the primer pairs and calibrated against the Ct obtained with gDNA, which represented the number of target genes in the corresponding parasite genotype. The following formula was used to calculate relative expression values (RELATEXP):
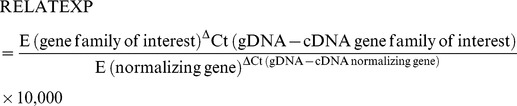



Parasites cultivated separately in two culture dishes were analyzed at least in duplicate and the resulting mean ΔCt value or relative expression value was plotted with standard deviation against the time of cultivation and parasite stage, determined as the most dominant stage by Giemsa staining of a corresponding sample.

### Cloning, Sequencing and Analysis of PCR Products

PCR conditions and the primers used to identify individual gene variants in each expression peak have been previously described [26]. Amplicons were purified by gel extraction using a NucleoSpin Extract II Kit (Macherey-Nagel) and cloned into the pCR2.1 TOPO vector, and then the vector was used to transform TOP10 cells (TOPO TA Cloning Kit; Invitrogen). Inserts were sequenced using the M13 primer set (AGOWA GmbH, Berlin) and analyzed with MacVector 10.0.2 software.

### Statistical Tests

To evaluate potentially biased amplification, the proportions of the amplified gene variants in the gDNA samples were compared with the expected proportions according to a binomial distribution. It was assumed that every *P. falciparum* genotype possessed 102 *rif-A*, 41 *rif-B*, 32 *stevor*, and 13 *pfmc-2tm* genes. The *P* values were corrected according to Bonferroni for the number of clones analyzed by PCR.

To analyze differences in gene expression, cloned transcripts were randomly selected from agar plates and the corresponding sequences identified. The proportions of the expressed gene variants were compared between different parasite stages and generations in contingency tables using c^2^ tests. *P* values were corrected according to Bonferroni for the number of clones analyzed. Furthermore, the proportions of the expressed sequences from the different time points were compared in contingency tables using c^2^ tests and the sequences obtained from gDNA-amplification. *P* values were corrected according to Bonferroni for the number of clones analyzed in paired samples.

### Generation of Antisera

The α-RIF40 (AF483820: AA35-215) [42] and α-*Pf*MC-2TM-SC (PFF1525c: AA 54–159) antisera were raised in rats or mice against recombinant proteins generated using Gb21 or 3D7 genomic DNA. The α-*Pf*MC-2TM-CT antiserum was generated by immunizing rabbits with the peptide FKKLKTKLNTFFQNKKQITK from the 3D7 variant PFF1525c (amino acids: 212–231). Immunizations of rats and rabbits were performed by Pineda Antikörper-Service, Berlin, Germany. The following additional antibodies were also used: rat α-RIF29n, rat α-RIF44, and rat α-RIF50 antisera (provided by Mo-Quen Klinkert) [42]; mouse α-STEVOR (α-PFA0750w, α-MAL13P1.7, α-PFL2610w and α-PFC0025c) (provided by Nadine Schreiber) [43]; rabbit α-MSP1, rabbit α-BiP, and rabbit α-EBA175 (provided by Klemens Engelberg and Tim-Wolf Gilberger); mouse and rat α-SBP1 (provided by Catherine Braun-Breton); rabbit α-spectrin (Sigma-Aldrich; S1515); and monoclonal mouse α-ATS antibody 6H1 [44]. All α-STEVOR sera were mixed in equal amounts for IFA analyses and the mixture was referred to as α-STEVOR-mix. As a positive control, serum was obtained from a semi-immune African with high titer antibodies to *P. falciparum* antigen [26]. Specific reactivity of the newly synthesized rat α-RIF40, mouse α-*Pf*MC-2TM-SC and rabbit α-*Pf*MC-2TM-CT sera for RIFIN and *Pf*MC-2TM proteins was determined by immunoblot analysis using membrane fractions from different *P. falciparum* isolates (Fig. S1).

### Immunoblot Analysis

Soluble proteins and hemoglobin were released from IEs by saponin lysis and the remaining pellet was lysed in 2×SDS sample buffer at a concentration of 1×10^6^ cells µl^−1^. Merozoites were isolated using a protocol adopted from Boyle *et al.* [69]. Lysate from 1×10^7^ mature stage or 7.5×10^7^ merozoites was separated on 6% to 12% Bis-Tris or tricine gels by SDS-PAGE and then analyzed by immunoblot according to standard procedures. Antisera were diluted and used for immunoblot analyses as follows: rat α-RIF40 1:2.000, rat α-RIF44 1:2.000, rat α-RIF50 1:2.000, mouse α-STEVOR-mix and individual α-STEVOR sera (α-PFA0750w, α-MAL13P1.7, α-PFL2610w and α-PFC0025c) 1∶3.000, mouse α-*Pf*MC-2TM-SC 1∶4.000, rabbit α-*Pf*MC-2TM-CT 1∶2.000, rabbit α-BiP 1∶10.000, and rabbit α-EBA175 1:2.000. Horseradish peroxidase-coupled secondary antibody (Dianova) was detected by chemiluminescence and visualized on Hyperfilm-ECL (Amersham).

### IFA

Smears were air-dried and fixed for 5 min with 100% methanol at −20°C. After rehydration for 10 min in PBS, the slides were incubated for 2 h at room temperature with the indicated antisera diluted in PBS/1% BSA. For co-localisation studies, further antisera were processed similarly following incubation of the VSA-specific antibody. After washing three times with PBS, the slides were incubated with Alexa488- or Alexa594-coupled α-mouse, α-rat, α-rabbit or α-human secondary antibodies (1∶400 dilution; Invitrogen) and DAPI (1 µg ml^−1^; Sigma). After repeated washing in PBS, the slides were mounted with MOWIOL 4–88 (Calbiochem) and then viewed through a 100X oil immersion lens on a UV-equipped Leica DM RB microscope. Antisera were diluted as follows: α-ATS antibody 6H1 1:50, rat α-RIF29n 1∶100, rat α-RIF40 1:300, mouse α-STEVOR-mix 1∶300, mouse α-*Pf*MC-2TM-SC 1∶300, rabbit α-*Pf*MC-2TM-CT 1∶200, rabbit α-spectrin 1∶200, mouse and rat α-SBP1 1:300 and rabbit α-MSP1 1:4.000. The proportion of positively-stained cells as well as VSA localization was quantified by counting at least 100 IEs stained with the corresponding α-VSA sera solely. As a control, no fluorescence signals were observed with secondary antibody alone and pre-immune sera. Pre-immune sera from animals immunized with recombinant RIF40 and the *Pf*MC-2TM-CT peptide showed faint, diffuse background fluorescence in intracellular parasites.

### DNA Purification and Genotyping

Genomic DNA was isolated from ficoll-purified *ex vivo* erythrocytes as well as from cultivated parasites using the QIAamp DNA Blood Mini Kit (Qiagen). To determine the number of *P. falciparum* genotypes in the *ex vivo* blood samples, MSP1 genotyping was carried out as described elsewhere [70].

## Supporting Information

Figure S1 **Immunoblot analysis of the specific reactivity of the antisera used for the detection of VSAs.** Immunoblot analysis of membrane extracts from pigmented parasite stages using the indicated antisera. Analysis of the negative control uninfected erythrocytes (RBC; lane 1) confirmed the absence of any non-specific reactivity with erythrocyte membrane proteins. Analysis of strain 3D7 and clinical isolates #1, #3 and #4 revealed protein bands of the correct size corresponding to the indicated protein families. Coomassie staining of the gel demonstrated similar amounts of protein loaded onto each lane.
(TIF)

Figure S2 **Sequences of transcribed** ***2tm*** **genes in different parasite stages of isolates #1–#4 and strain 3D7.** Amplification was carried out using universal working primers that spanned a longer and more variable region than the primers used for real-time PCR. To control for potential primer bias towards preferentially amplified genes, gDNA was analyzed in parallel. Amplicons were cloned and 15 bacterial colonies on average were sequenced to identify the expressed genes. Statistical analysis revealed no apparent differences between the genes expressed in ring stages and trophozoites. The star indicates sequences with significantly different levels between cDNA and gDNA in the respective *P. falciparum* isolate. *P*<0.05 (*); *P*<0.01 (**); *P*<0.001 (***).
(TIF)

Figure S3 **IFA and quantification of protein localization in isolates #1–4 and 3D7 using immune serum from a semi-immune patient.** IFA of different parasitic stages, as determined by time of *in vitro* cultivation (h) using immune serum obtained from a semi-immune patient [26] (**A**). Fluorescence signals and localization was quantified by visual scoring of at least 100 infected erythrocytes stained with the immune serum (**B**). Shown is the percentage of protein associated with the erythrocyte membrane (EM, blue), Maurer’s clefts (MC, violet), parasite membrane and parasitophorous vacuole membrane complex (PM/PVM, red), and inside the parasitic boundary (parasite, grey); cells that lacked specific fluorescence signals are also shown (negative, white). The summary percentage of all location sites is greater than 100 because some proteins localized to multiple sites within one cell. n.d.: not determined.
(TIF)

Figure S4 **IFA and quantification of VSA localization in isolates #1–4 and 3D7 using the α-ATS antiserum.** IFA of different parasitic stages, as determined by time of *in vitro* cultivation (h) using an antiserum directed against the C-terminal domain of *Pf*EMP1 (α-ATS) (**A**). Fluorescence signals and localization was quantified by visual scoring of at least 100 infected erythrocytes stained with the α-ATS serum (**B**). Shown is the percentage of protein associated with the erythrocyte membrane (EM, blue), Maurer’s clefts (MC, violet), parasite membrane and parasitophorous vacuole membrane complex (PM/PVM, red), and inside the parasitic boundary (parasite, grey); cells that lacked specific fluorescence signals are also shown (negative, white). The summary percentage of all location sites is greater than 100 because some proteins localized to multiple sites within one cell. n.d.: not determined.
(TIF)

Figure S5 **IFA and quantification of VSA localization in isolates #1–4 and 3D7 using the α-RIF29n antiserum.** IFA of different parasitic stages, as determined by time of *in vitro* cultivation (h) using an antiserum directed against A-type RIFIN proteins (α-RIF29n) (**A**). Fluorescence signals and localization was quantified by visual scoring of at least 100 infected erythrocytes stained with the α-RIF29n serum (**B**). Shown is the percentage of protein associated with the erythrocyte membrane (EM, blue), Maurer’s clefts (MC, violet), parasite membrane and parasitophorous vacuole membrane complex (PM/PVM, red), and inside the parasitic boundary (parasite, grey); cells that lacked specific fluorescence signals are also shown (negative, white). The summary percentage of all location sites is greater than 100 because some proteins localized to multiple sites within one cell. n.d.: not determined.
(TIF)

Figure S6 **IFA and quantification of VSA localization in isolates #1–4 and 3D7 using the α-RIF40 antiserum.** IFA of different parasitic stages, as determined by time of *in vitro* cultivation (h) using an antiserum directed against A-type RIFIN proteins (α-RIF40) (**A**). Fluorescence signals and localization was quantified by visual scoring of at least 100 infected erythrocytes stained with the α-RIF40 serum (**B**). Shown is the percentage of protein associated with the erythrocyte membrane (EM, blue), Maurer’s clefts (MC, violet), parasite membrane and parasitophorous vacuole membrane complex (PM/PVM, red), and inside the parasitic boundary (parasite, grey); cells that lacked specific fluorescence signals are also shown (negative, white). The summary percentage of all location sites is greater than 100 because some proteins localized to multiple sites within one cell. n.d.: not determined.
(TIF)

Figure S7 **IFA and quantification of VSA localization in isolates #1–4 and 3D7 using a mixture of α-STEVOR antisera.** IFA of different parasitic stages, as determined by time of *in vitro* cultivation (h) using an antisera mixture directed against different STEVOR variants (α-STEVOR-mix) (**A**). Fluorescence signals and localization was quantified by visual scoring of at least 100 infected erythrocytes stained with the α-STEVOR-mix (**B**). Shown is the percentage of protein associated with the erythrocyte membrane (EM, blue), Maurer’s clefts (MC, violet), parasite membrane and parasitophorous vacuole membrane complex (PM/PVM, red), and inside the parasitic boundary (parasite, grey); cells that lacked specific fluorescence signals are also shown (negative, white). The summary percentage of all location sites is greater than 100 because some proteins localized to multiple sites within one cell. n.d.: not determined.
(TIF)

Figure S8 **IFA and quantification of VSA localization in isolates #1–4 and 3D7 using the α-** ***Pf*** **MC-2TM-SC antiserum.** IFA of different parasitic stages, as determined by time of *in vitro* cultivation (h) using an antiserum directed against the semi-conserved domain of *Pf*MC-2TM proteins (α-*Pf*MC-2TM-SC) (**A**). Fluorescence signals and localization was quantified by visual scoring of at least 100 infected erythrocytes stained with the α-*Pf*MC-2TM-SC serum (**B**). Shown is the percentage of protein associated with the erythrocyte membrane (EM, blue), Maurer’s clefts (MC, violet), parasite membrane and parasitophorous vacuole membrane complex (PM/PVM, red), and inside the parasitic boundary (parasite, grey); cells that lacked specific fluorescence signals are also shown (negative, white). The summary percentage of all location sites is greater than 100 because some proteins localized to multiple sites within one cell. n.d.: not determined.
(TIF)

Figure S9 **IFA and quantification of VSA localization in isolates #1–4 and 3D7 using the α-** ***Pf*** **MC-2TM-CT antiserum.** IFA of different parasitic stages, as determined by time of *in vitro* cultivation (h) using an antiserum directed against the C-terminal domain of *Pf*MC-2TM proteins (α-*Pf*MC-2TM-CT) (**A**). Fluorescence signals and localization was quantified by visual scoring of at least 100 infected erythrocytes stained with the α-*Pf*MC-2TM-CT serum (**B**). Shown is the percentage of protein associated with the erythrocyte membrane (EM, blue), Maurer’s clefts (MC, violet), parasite membrane and parasitophorous vacuole membrane complex (PM/PVM, red), and inside the parasitic boundary (parasite, grey); cells that lacked specific fluorescence signals are also shown (negative, white). The summary percentage of all location sites is greater than 100 because some proteins localized to multiple sites within one cell. n.d.: not determined.
(TIF)

Figure S10 **Immunoblot analysis of RIFIN and STEVOR in isolated merozoites.** The presence of STEVOR and RIFIN proteins in isolated merozoites from strain 3D7 and clinical isolates #1, #3 and #4 was confirmed by immunoblot analysis. For the detection of RIFIN, α-RIF40 and α-RIF50 antisera were used; for the detection of STEVOR proteins in merozoites, α-PFL2610w, α-PFC0025c, α-MAL13P1.7, and α-PFA0750w antisera were used. The α-EBA175 antiserum was used as a positive control. No signals were obtained using α-spectrin, α-glycophorin A/B and α-*Pf*EMP1 (αATS) antisera, confirming the absence of erythrocytic membranes in the merozoite fractions (data not shown). Coomassie staining of the gel confirmed equal loading of each lane with approximately 7.5×10^7^ merozoites.
(TIF)

Figure S11 **Co-localization of VSAs during the intraerythrocytic developmental cycle. A, B:** Co-localization of *Pf*EMP1, RIFIN, STEVOR and *Pf*MC-2TM (green) with marker proteins for the erythrocyte membrane (spectrin), the Maurer’s clefts (SBP1) and the merozoite surrounding membrane (MSP1) (red). Subcellular VSA localization was determined in trophozoites (**A**) as well as in schizonts and free merozites (**B**) from clinical isolates as well as strain 3D7. Nuclei were stained with DAPI (blue).
(TIF)

Table S1 **Evaluation of real-time PCR primer pairs targeting the** ***var*** **,** ***stevor*** **and** ***pfmc-2tm*** **gene families using genomic DNA from strain 3D7: results of cloning and sequencing of amplicons.**
(DOC)

Table S2 **Amplification efficiencies of the degenerate primer pairs used for quantitative real-time PCR: template dilution of genomic DNA (gDNA) or application of the DART algorithm (cDNA).**
(DOC)

Table S3 **Transcripts and sequences of** ***rif-A*** **,** ***rif-B*** **,** ***stevor*** **and** ***pfmc-2tm*** **genes in** ***P. falciparum*** **isolates #1, #2, #3 and #4 and strain 3D7 during the two peaks of expression in ring and trophozoite stages.**
(DOC)

Table S4 **Patient and isolate characteristics.**
(DOC)
