# Supplementary figures and images for: Temporal Expression and Localization Patterns of Variant Surface Antigens in Clinical Plasmodium falciparum Isolates during Erythrocyte Schizogony
Source: PLoS One. 2012 Nov 15;7(11):e49540. doi: 10.1371/journal.pone.0049540 (PMC3499489; doi:10.1371/journal.pone.0049540)

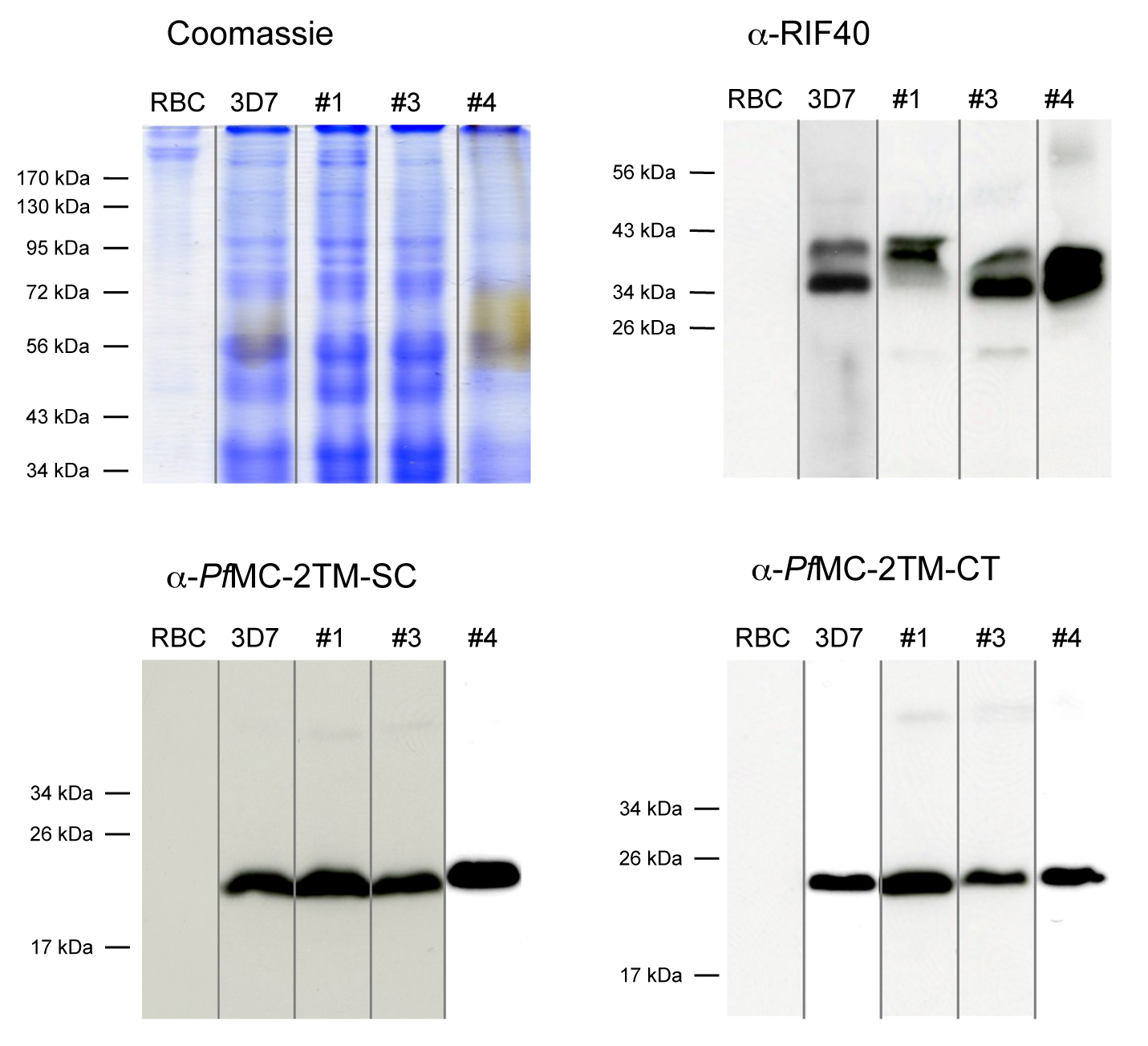

Supplement: Figure S1 — Immunoblot analysis of the specific reactivity of the antisera used for the detection of VSAs. Immunoblot analysis of membrane extracts from pigmented parasite stages using the indicated antisera. Analysis of the negative control uninfected erythrocytes (RBC; lane 1) confirmed the absence of any non-specific reactivity with erythrocyte membrane proteins. Analysis of strain 3D7 and clinical isolates #1, #3 and #4 revealed protein bands of the correct size corresponding to the indicated protein families. Coomassie staining of the gel demonstrated similar amounts of protein loaded onto each lane. (TIF) [file pone.0049540.s001.tif]

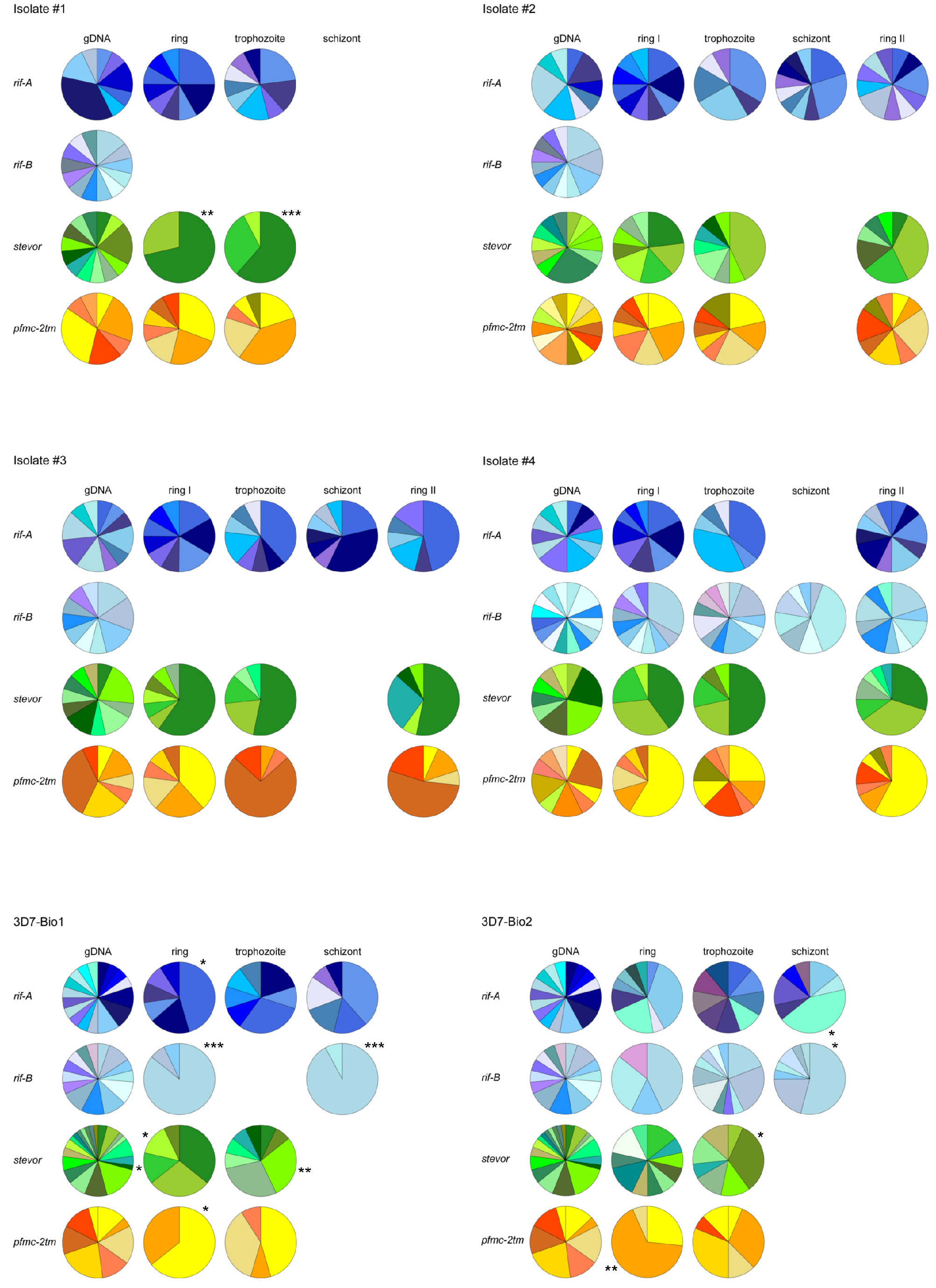

Supplement: Figure S2 — Sequences of transcribed 2tm genes in different parasite stages of isolates #1–#4 and strain 3D7. Amplification was carried out using universal working primers that spanned a longer and more variable region than the primers used for real-time PCR. To control for potential primer bias towards preferentially amplified genes, gDNA was analyzed in parallel. Amplicons were cloned and 15 bacterial colonies on average were sequenced to identify the expressed genes. Statistical analysis revealed no apparent differences between the genes expressed in ring stages and trophozoites. The star indicates sequences with significantly different levels between cDNA and gDNA in the respective P. falciparum isolate. P<0.05 (*); P<0.01 (**); P<0.001 (***). (TIF) [file pone.0049540.s002.tif]

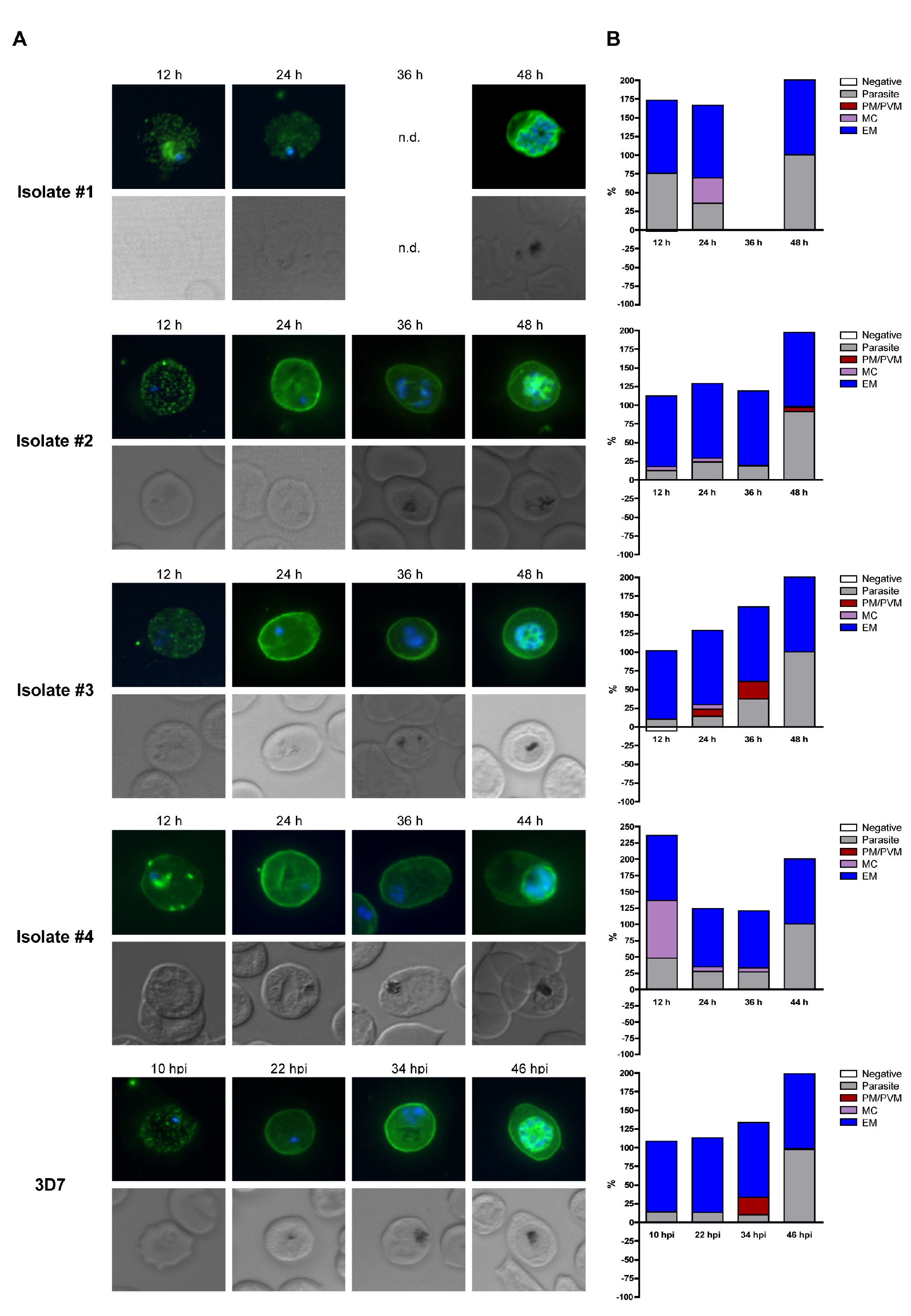

Supplement: Figure S3 — IFA and quantification of protein localization in isolates #1–4 and 3D7 using immune serum from a semi-immune patient. IFA of different parasitic stages, as determined by time of in vitro cultivation (h) using immune serum obtained from a semi-immune patient [26] (A). Fluorescence signals and localization was quantified by visual scoring of at least 100 infected erythrocytes stained with the immune serum (B). Shown is the percentage of protein associated with the erythrocyte membrane (EM, blue), Maurer’s clefts (MC, violet), parasite membrane and parasitophorous vacuole membrane complex (PM/PVM, red), and inside the parasitic boundary (parasite, grey); cells that lacked specific fluorescence signals are also shown (negative, white). The summary percentage of all location sites is greater than 100 because some proteins localized to multiple sites within one cell. n.d.: not determined. (TIF) [file pone.0049540.s003.tif]

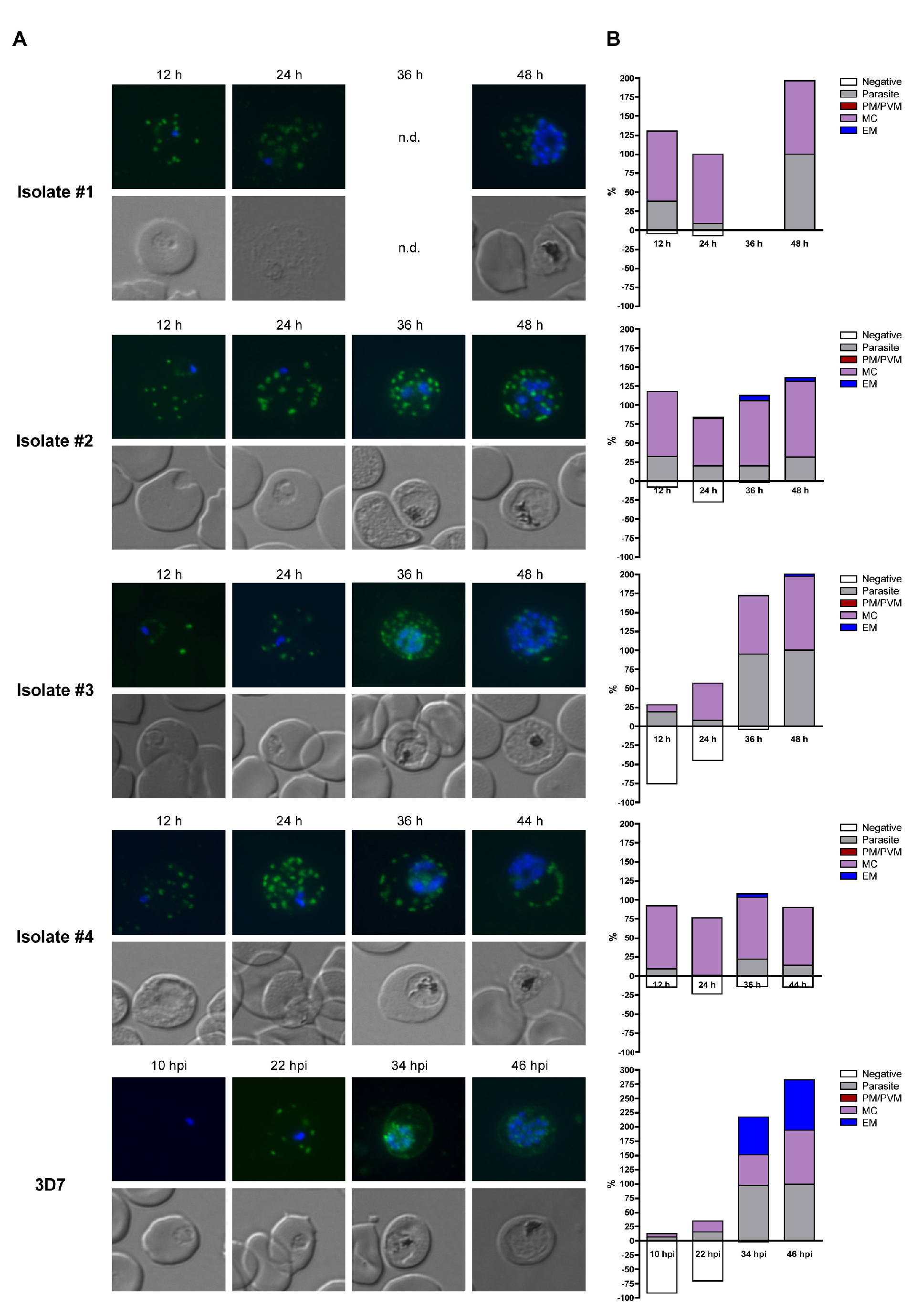

Supplement: Figure S4 — IFA and quantification of VSA localization in isolates #1–4 and 3D7 using the α-ATS antiserum. IFA of different parasitic stages, as determined by time of in vitro cultivation (h) using an antiserum directed against the C-terminal domain of PfEMP1 (α-ATS) (A). Fluorescence signals and localization was quantified by visual scoring of at least 100 infected erythrocytes stained with the α-ATS serum (B). Shown is the percentage of protein associated with the erythrocyte membrane (EM, blue), Maurer’s clefts (MC, violet), parasite membrane and parasitophorous vacuole membrane complex (PM/PVM, red), and inside the parasitic boundary (parasite, grey); cells that lacked specific fluorescence signals are also shown (negative, white). The summary percentage of all location sites is greater than 100 because some proteins localized to multiple sites within one cell. n.d.: not determined. (TIF) [file pone.0049540.s004.tif]

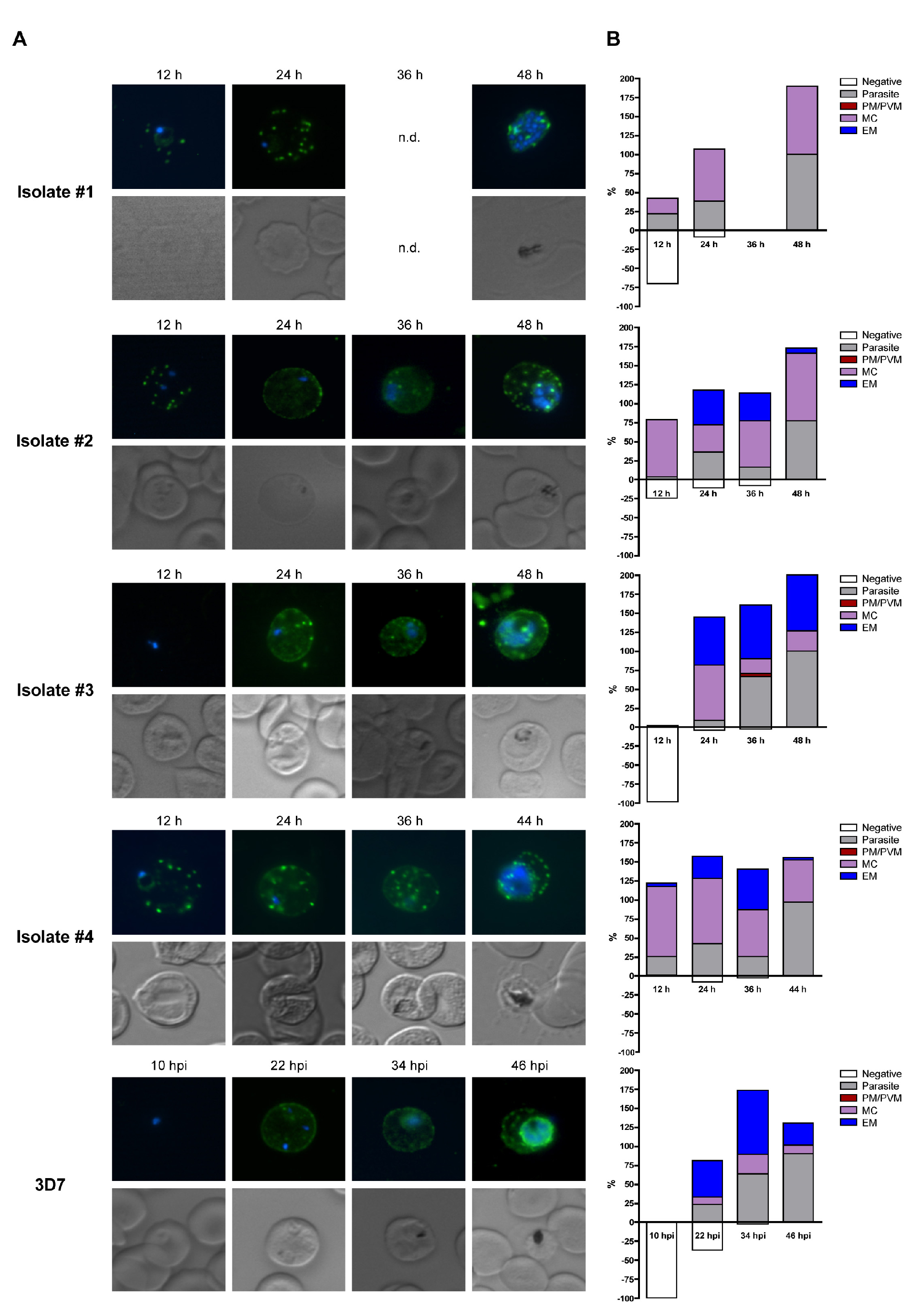

Supplement: Figure S5 — IFA and quantification of VSA localization in isolates #1–4 and 3D7 using the α-RIF29n antiserum. IFA of different parasitic stages, as determined by time of in vitro cultivation (h) using an antiserum directed against A-type RIFIN proteins (α-RIF29n) (A). Fluorescence signals and localization was quantified by visual scoring of at least 100 infected erythrocytes stained with the α-RIF29n serum (B). Shown is the percentage of protein associated with the erythrocyte membrane (EM, blue), Maurer’s clefts (MC, violet), parasite membrane and parasitophorous vacuole membrane complex (PM/PVM, red), and inside the parasitic boundary (parasite, grey); cells that lacked specific fluorescence signals are also shown (negative, white). The summary percentage of all location sites is greater than 100 because some proteins localized to multiple sites within one cell. n.d.: not determined. (TIF) [file pone.0049540.s005.tif]

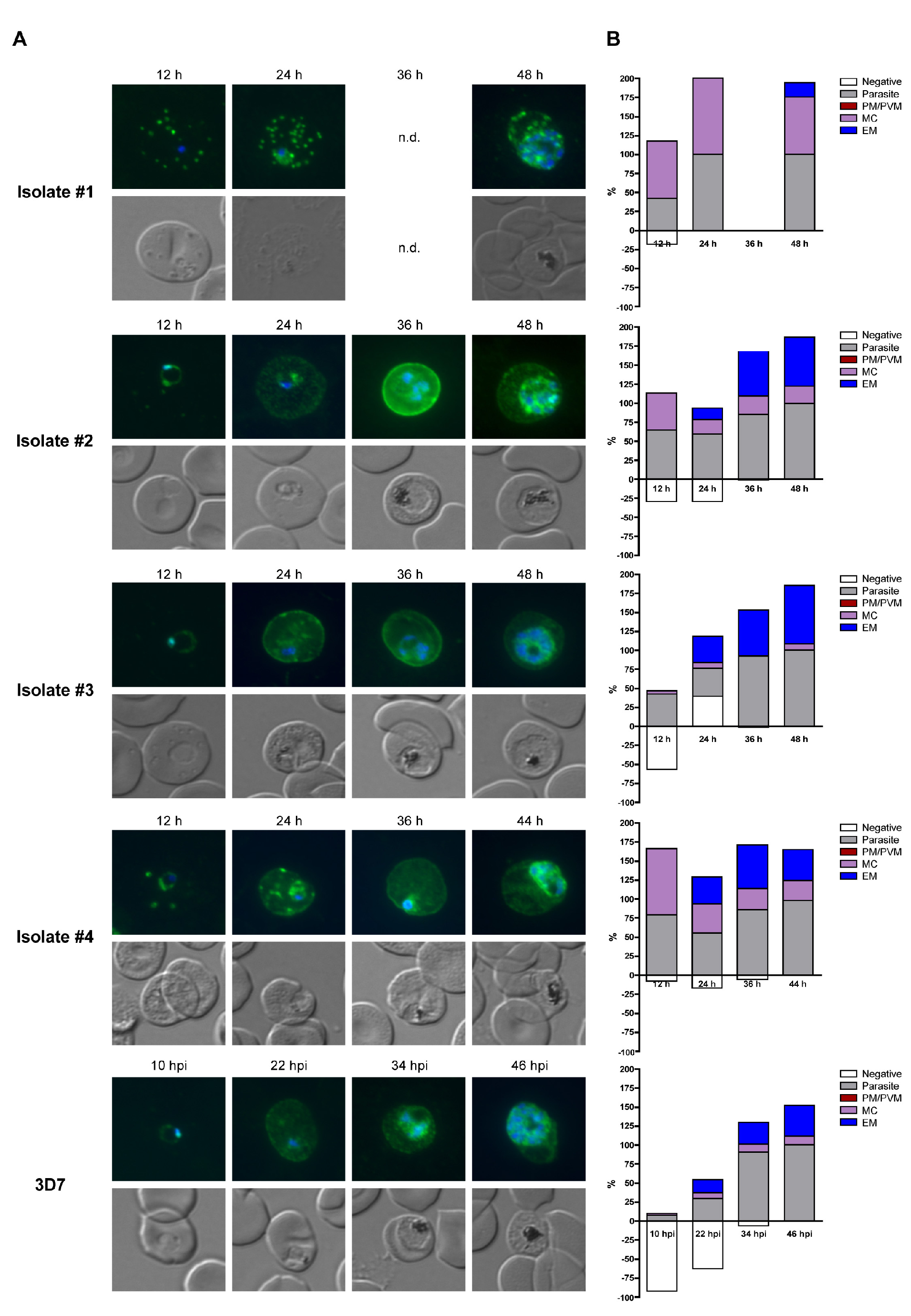

Supplement: Figure S6 — IFA and quantification of VSA localization in isolates #1–4 and 3D7 using the α-RIF40 antiserum. IFA of different parasitic stages, as determined by time of in vitro cultivation (h) using an antiserum directed against A-type RIFIN proteins (α-RIF40) (A). Fluorescence signals and localization was quantified by visual scoring of at least 100 infected erythrocytes stained with the α-RIF40 serum (B). Shown is the percentage of protein associated with the erythrocyte membrane (EM, blue), Maurer’s clefts (MC, violet), parasite membrane and parasitophorous vacuole membrane complex (PM/PVM, red), and inside the parasitic boundary (parasite, grey); cells that lacked specific fluorescence signals are also shown (negative, white). The summary percentage of all location sites is greater than 100 because some proteins localized to multiple sites within one cell. n.d.: not determined. (TIF) [file pone.0049540.s006.tif]

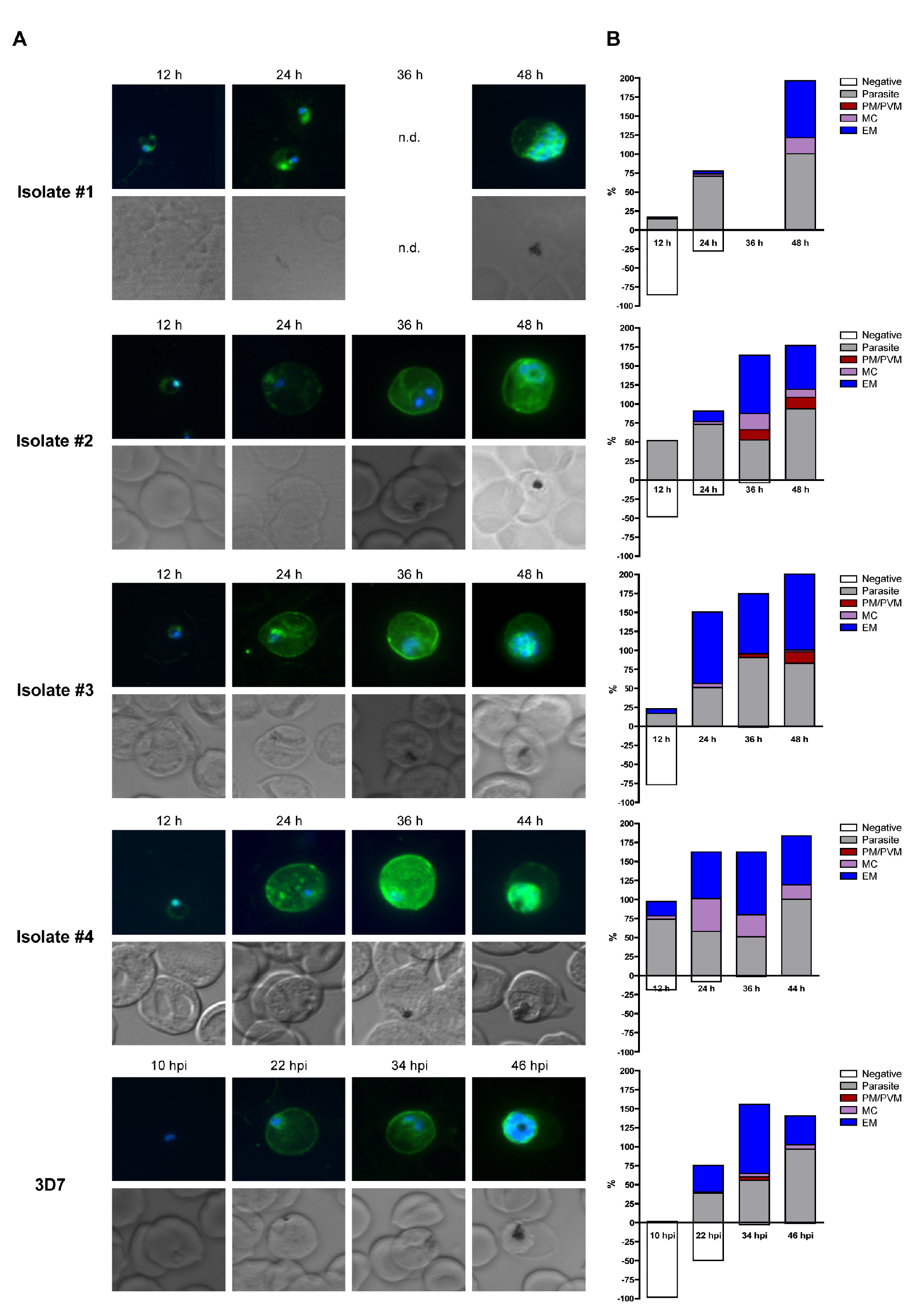

Supplement: Figure S7 — IFA and quantification of VSA localization in isolates #1–4 and 3D7 using a mixture of α-STEVOR antisera. IFA of different parasitic stages, as determined by time of in vitro cultivation (h) using an antisera mixture directed against different STEVOR variants (α-STEVOR-mix) (A). Fluorescence signals and localization was quantified by visual scoring of at least 100 infected erythrocytes stained with the α-STEVOR-mix (B). Shown is the percentage of protein associated with the erythrocyte membrane (EM, blue), Maurer’s clefts (MC, violet), parasite membrane and parasitophorous vacuole membrane complex (PM/PVM, red), and inside the parasitic boundary (parasite, grey); cells that lacked specific fluorescence signals are also shown (negative, white). The summary percentage of all location sites is greater than 100 because some proteins localized to multiple sites within one cell. n.d.: not determined. (TIF) [file pone.0049540.s007.tif]

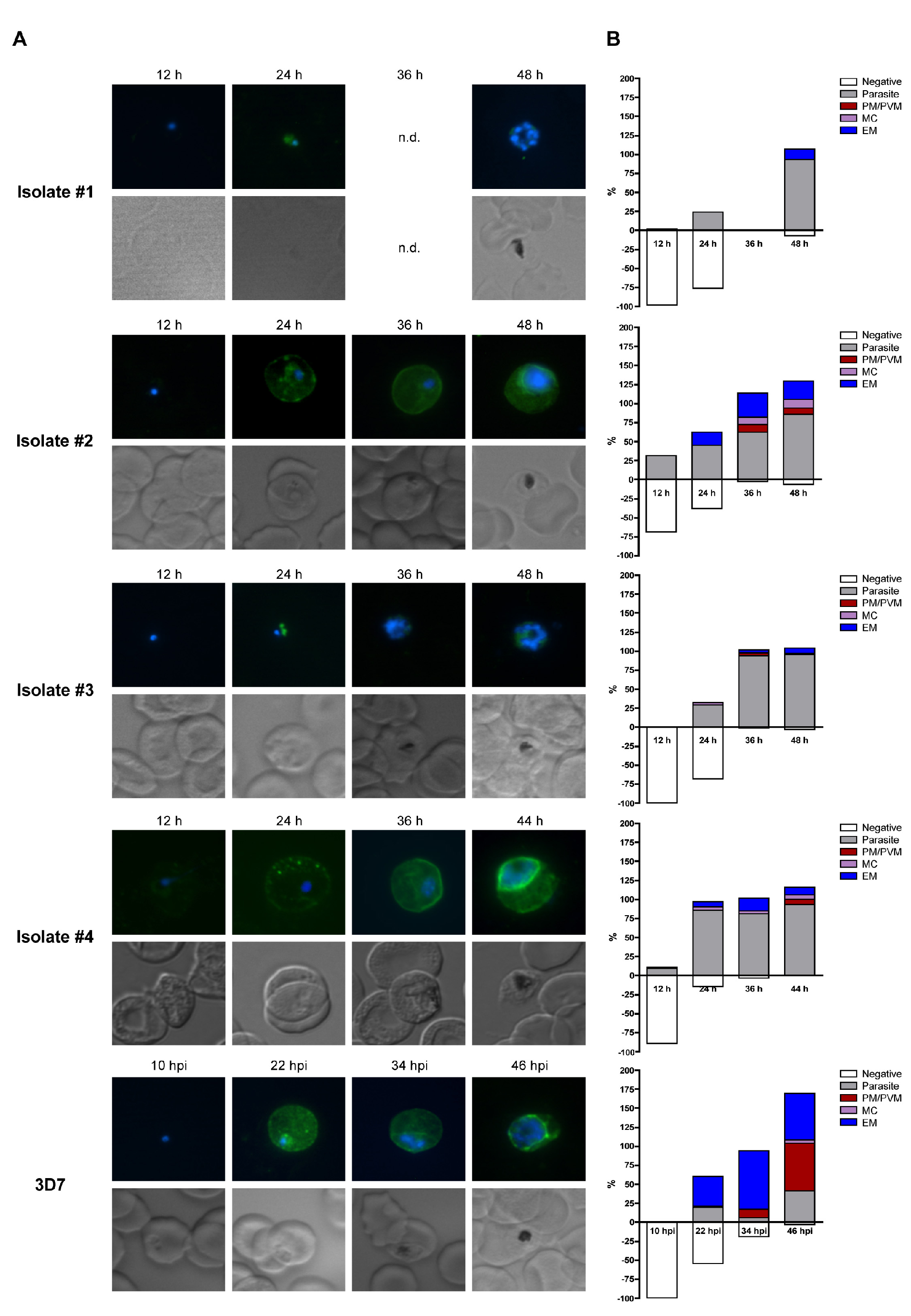

Supplement: Figure S8 — IFA and quantification of VSA localization in isolates #1–4 and 3D7 using the α- Pf MC-2TM-SC antiserum. IFA of different parasitic stages, as determined by time of in vitro cultivation (h) using an antiserum directed against the semi-conserved domain of PfMC-2TM proteins (α-PfMC-2TM-SC) (A). Fluorescence signals and localization was quantified by visual scoring of at least 100 infected erythrocytes stained with the α-PfMC-2TM-SC serum (B). Shown is the percentage of protein associated with the erythrocyte membrane (EM, blue), Maurer’s clefts (MC, violet), parasite membrane and parasitophorous vacuole membrane complex (PM/PVM, red), and inside the parasitic boundary (parasite, grey); cells that lacked specific fluorescence signals are also shown (negative, white). The summary percentage of all location sites is greater than 100 because some proteins localized to multiple sites within one cell. n.d.: not determined. (TIF) [file pone.0049540.s008.tif]

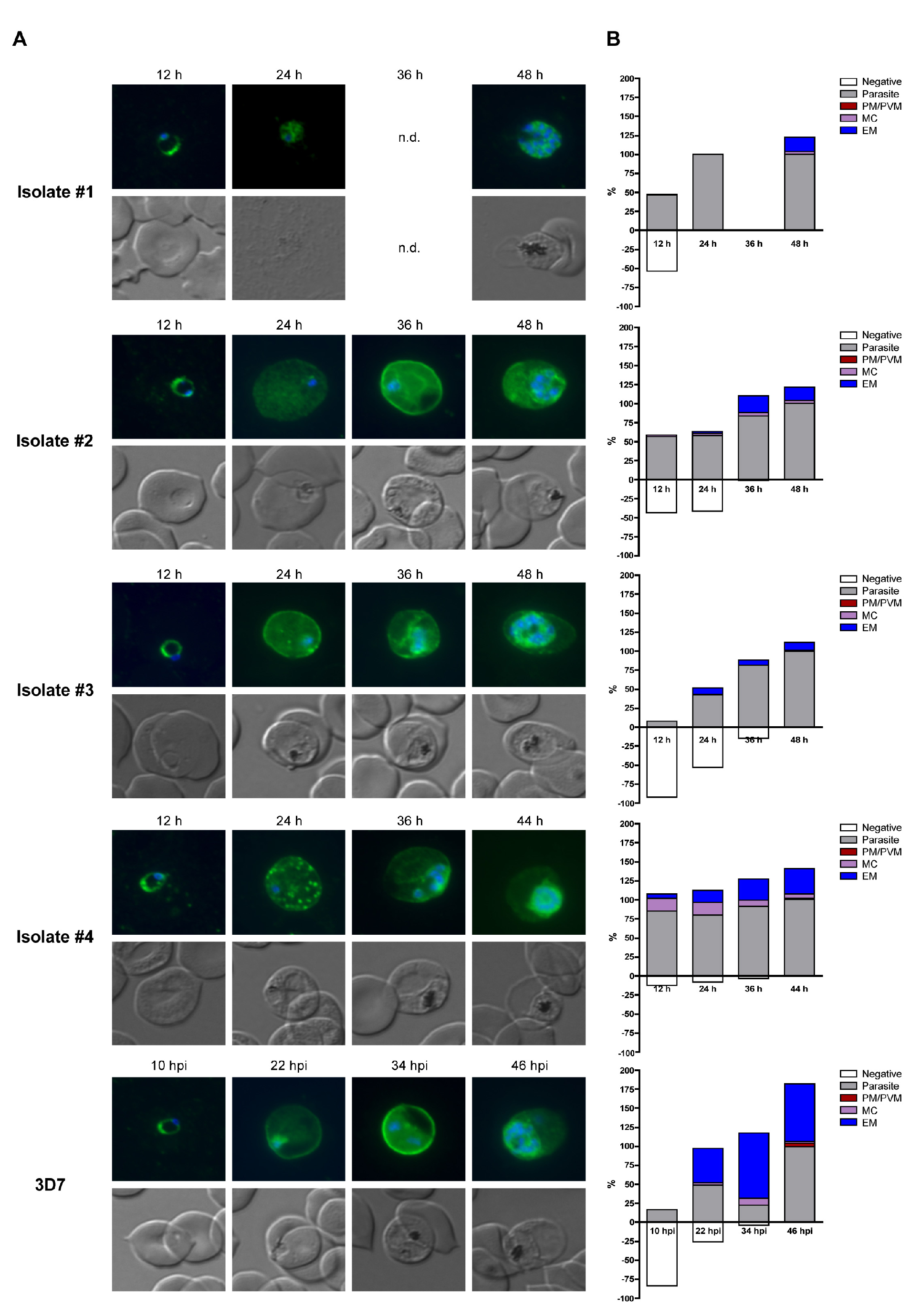

Supplement: Figure S9 — IFA and quantification of VSA localization in isolates #1–4 and 3D7 using the α- Pf MC-2TM-CT antiserum. IFA of different parasitic stages, as determined by time of in vitro cultivation (h) using an antiserum directed against the C-terminal domain of PfMC-2TM proteins (α-PfMC-2TM-CT) (A). Fluorescence signals and localization was quantified by visual scoring of at least 100 infected erythrocytes stained with the α-PfMC-2TM-CT serum (B). Shown is the percentage of protein associated with the erythrocyte membrane (EM, blue), Maurer’s clefts (MC, violet), parasite membrane and parasitophorous vacuole membrane complex (PM/PVM, red), and inside the parasitic boundary (parasite, grey); cells that lacked specific fluorescence signals are also shown (negative, white). The summary percentage of all location sites is greater than 100 because some proteins localized to multiple sites within one cell. n.d.: not determined. (TIF) [file pone.0049540.s009.tif]

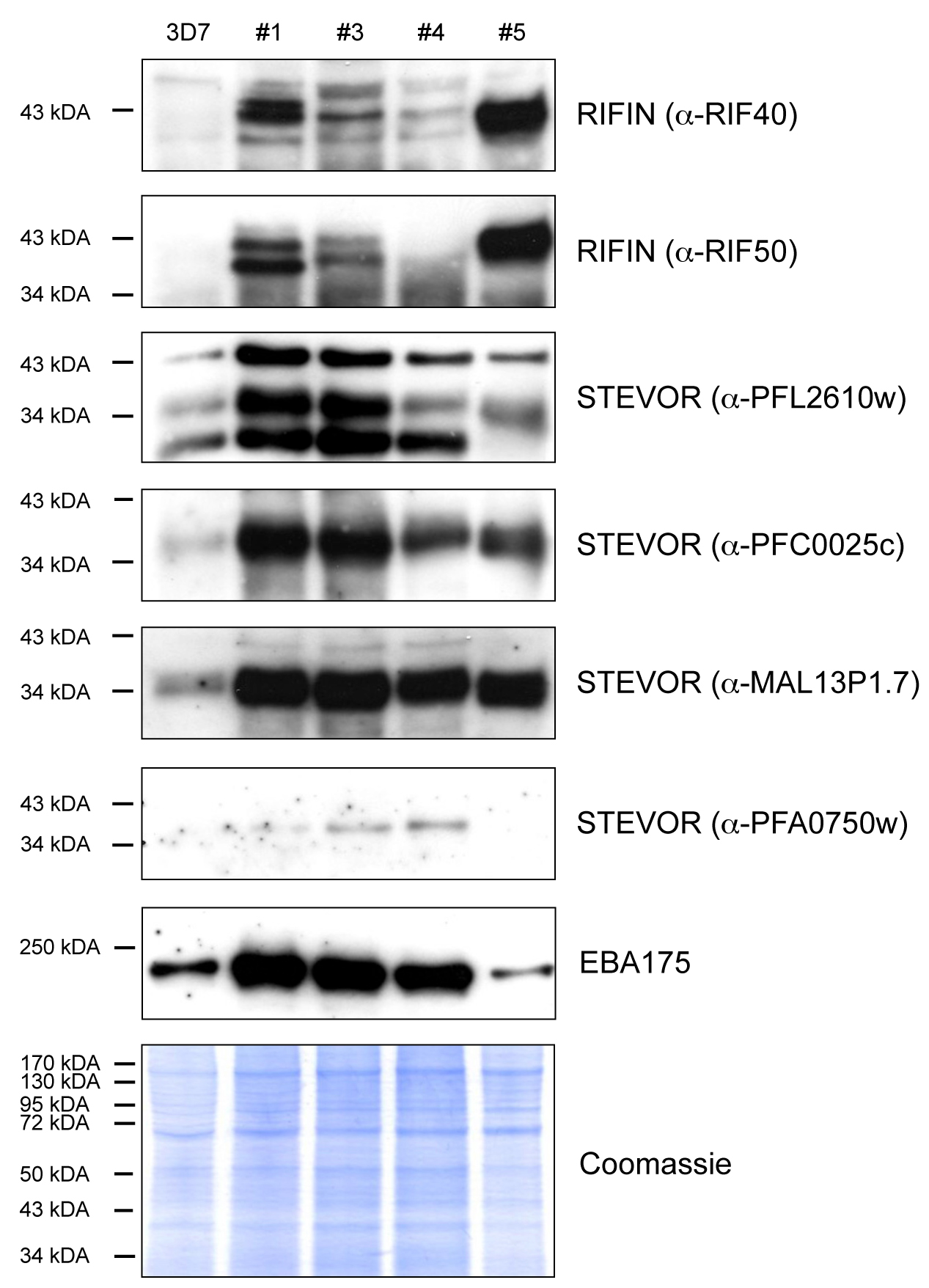

Supplement: Figure S10 — Immunoblot analysis of RIFIN and STEVOR in isolated merozoites. The presence of STEVOR and RIFIN proteins in isolated merozoites from strain 3D7 and clinical isolates #1, #3 and #4 was confirmed by immunoblot analysis. For the detection of RIFIN, α-RIF40 and α-RIF50 antisera were used; for the detection of STEVOR proteins in merozoites, α-PFL2610w, α-PFC0025c, α-MAL13P1.7, and α-PFA0750w antisera were used. The α-EBA175 antiserum was used as a positive control. No signals were obtained using α-spectrin, α-glycophorin A/B and α-PfEMP1 (αATS) antisera, confirming the absence of erythrocytic membranes in the merozoite fractions (data not shown). Coomassie staining of the gel confirmed equal loading of each lane with approximately 7.5×10^7^ merozoites. (TIF) [file pone.0049540.s010.tif]

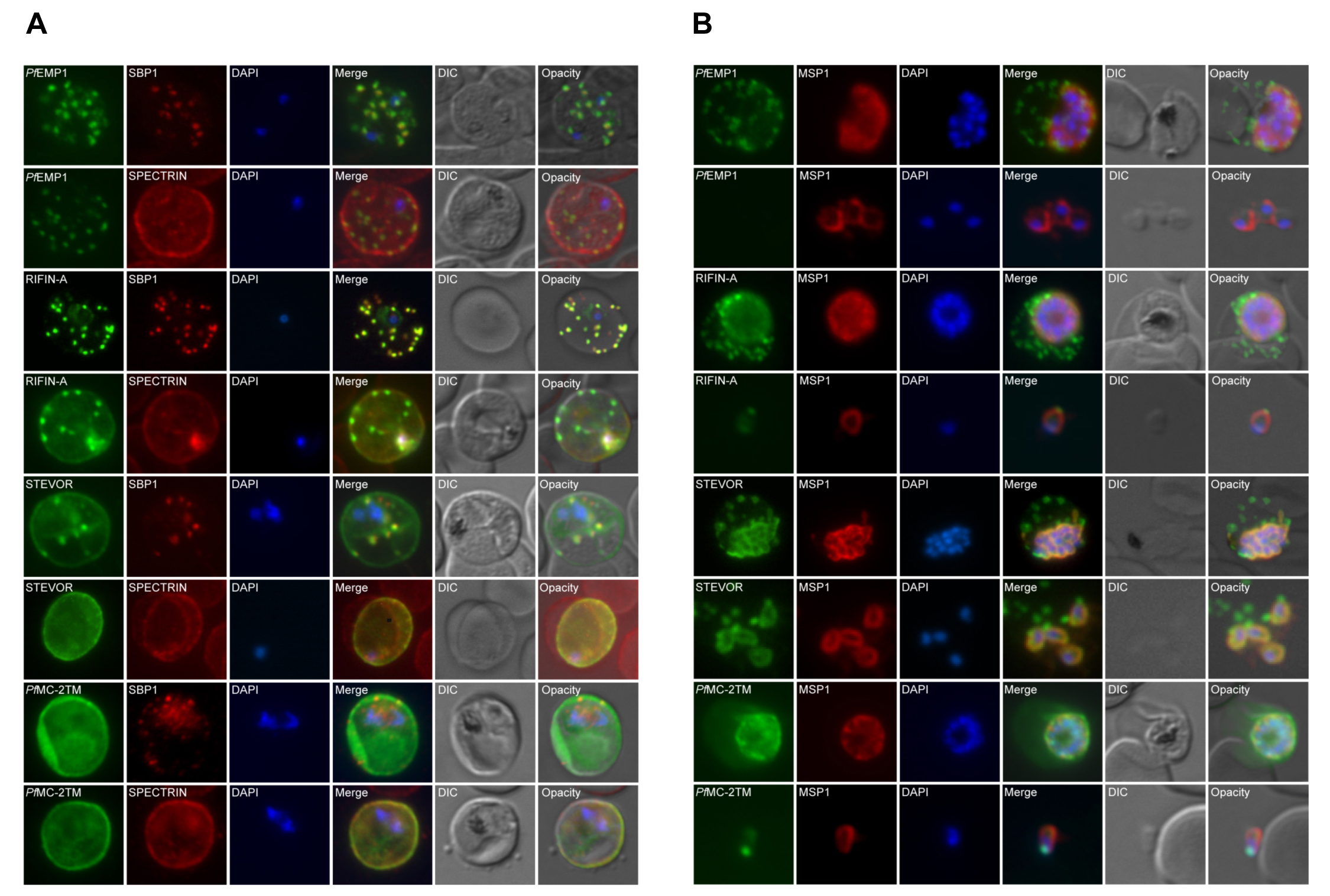

Supplement: Figure S11 — Co-localization of VSAs during the intraerythrocytic developmental cycle. A, B: Co-localization of PfEMP1, RIFIN, STEVOR and PfMC-2TM (green) with marker proteins for the erythrocyte membrane (spectrin), the Maurer’s clefts (SBP1) and the merozoite surrounding membrane (MSP1) (red). Subcellular VSA localization was determined in trophozoites (A) as well as in schizonts and free merozites (B) from clinical isolates as well as strain 3D7. Nuclei were stained with DAPI (blue). (TIF) [file pone.0049540.s011.tif]
