# Supplementary material for: Temporal Expression and Localization Patterns of Variant Surface Antigens in Clinical Plasmodium falciparum Isolates during Erythrocyte Schizogony
Source: PLoS One. 2012 Nov 15;7(11):e49540. doi: 10.1371/journal.pone.0049540 (PMC3499489; doi:10.1371/journal.pone.0049540)
Supplement: Table S1 — Evaluation of real-time PCR primer pairs targeting the var , stevor and pfmc-2tm gene families using genomic DNA from strain 3D7: results of cloning and sequencing of amplicons. (DOC) [file pone.0049540.s012.doc]

**Table S1: Evaluation of real-time PCR primer pairs targeting the *var*, *stevor* and *pfmc-2tm* gene families using genomic DNA from strain 3D7: results of cloning and sequencing of amplicons.**

| ***var*** | | | ***stevor*** | | | ***pfmc-2tm*** | | |
| --- | --- | --- | --- | --- | --- | --- | --- | --- |
| **N** | **Gene no.** | **%** | **N** | **Gene no.** | **%** | **N** | **Gene no.** | **%** |
| 18 | PFA0765c* | 16.7 | 33 | MAL8P1.217, PFC0025c, PF07_0130,  PFI0045c, PFA0090c, PFC1105c** | 54.6^#^ | 16 | PFA0680c, PFB0985c, PFF0060w,  PFF1525c | 50.0^#^ |
|  | PFD1005c* | 16.7 |  | MAL7P1.218 | 9.1 |  | PF11_0025, PF10_0390 | 18.8^#^ |
|  | PFL0005c | 11.1 |  | PFF1550w | 6.1 |  | MAL8P1.213, PFA0065w | 12.5^#^ |
|  | PFL1960w | 11.1 |  | PFL2610w | 6.1 |  | MAL7P1.58 | 6.3 |
|  | PFF1595c | 11.1 |  | PF10_0395 | 6.1 |  | PFC1080c | 6.3 |
|  | PFD0020c | 5.6 |  | PFD0065w | 3.0 |  | MAL7P1.5 | 6.3 |
|  | PF07_0050 | 5.6 |  | PFD0125c | 3.0 |  |  |  |
|  | PFL1955w | 5.6 |  | PF11_0516 | 3.0 |  |  |  |
|  | PFL0935c | 5.6 |  | PF14_0767 | 3.0 |  |  |  |
|  | PFB0010w | 5.6 |  | PFB0065w | 3.0 |  |  |  |
|  | PFC1015c | 5.6 |  | PFB0025c | 3.0 |  |  |  |

^#^ Listed genes cannot be discriminated due to the short region amplified.

* Significant bias P_c_ < 0.05.

** Significant bias P_c_ < 0.01.

N, number of clones sequenced.
