# Supplementary material for: Temporal Expression and Localization Patterns of Variant Surface Antigens in Clinical Plasmodium falciparum Isolates during Erythrocyte Schizogony
Source: PLoS One. 2012 Nov 15;7(11):e49540. doi: 10.1371/journal.pone.0049540 (PMC3499489; doi:10.1371/journal.pone.0049540)
Supplement: Table S2 — Amplification efficiencies of the degenerate primer pairs used for quantitative real-time PCR: template dilution of genomic DNA (gDNA) or application of the DART algorithm (cDNA). (DOC) [file pone.0049540.s013.doc]

**Table S2: Amplification efficiencies of degenerate primer pairs used for quantitative real-time PCR: template dilution of genomic DNA (gDNA) or application of the DART algorithm (cDNA).**

|  |  | **Template dilution (gDNA)** | | | **DART (cDNA)** | |
| --- | --- | --- | --- | --- | --- | --- |
|  |  | **Efficiency** | **M [ng]^1^** | **n^2^** | **Efficiency** | **n^3^** |
| **3D7** | ***fructose-bisphospate aldolase*** | 1.97 (±0.07) | 50–0.005 | 13 | 1.92 (±0.16) | 435 |
|  | ***var*** | 1.82 (±0.09) | 50–0.5 | 2 | 1.84 (±0.20) | 91 |
|  | ***rif-A*** | 1.74 | 50–0.05 | 1 | 1.76 (±0.11) | 154 |
|  | ***rif-B*** | 1.56 (±0.06) | 100–0.05 | 4 | 1.54 (±0.12) | 24 |
|  | ***stevor*** | 1.88 (±0.03) | 50–0.005 | 3 | 1.91 (±0.11) | 124 |
|  | ***pfmc-2tm*** | 1.85 (±0.05) | 50–0.0005 | 5 | 1.83 (±0.09) | 134 |
| **Isolate #1** | ***fructose-bisphospate aldolase*** | 1.99 (±0.02) | 75–0.5 | 2 | 1.94 (±0.10) | 118 |
|  | ***var*** | n.d. | | | 1.90 (±0.07) | 19 |
|  | ***rif-A*** |  |  |  | 1.85 (±0.06) | 11 |
|  | ***rif-B*** | 1.57 | 75–0.5 | 1 | n.d. | |
|  | ***stevor*** | 1.88 | 50–0.05 | 1 | 1.93 (±0.12) | 28 |
|  | ***pfmc-2tm*** | 1.86 | 75–0.05 | 1 | 1.82 (±0.11) | 37 |
| **Isolate #2** | ***fructose-bisphospate aldolase*** | n.d. | | | 1.90 (±0.15) | 248 |
|  | ***var*** |  |  |  | 1.91 (±0.15) | 41 |
|  | ***rif-A*** |  |  |  | 1.77 (±0.10) | 59 |
|  | ***rif-B*** |  |  |  | n.d. | |
|  | ***stevor*** |  |  |  | 1.92 (±0.12) | 51 |
|  | ***pfmc-2tm*** |  |  |  | 1.87 (±0.11) | 49 |
| **Isolate #3** | ***fructose-bisphospate aldolase*** | 2.00 (±0.04) | 50–0.05 | 2 | 1.92 (±0.17) | 257 |
|  | ***var*** | n.d. | | | 1.91 (±0.17) | 40 |
|  | ***rif-A*** |  |  |  | 1.74 (±0.09) | 55 |
|  | ***rif-B*** | 1.56 | 50–0.05 | 1 | n.d. | |
|  | ***stevor*** | n.d. | | | 1.94 (±0.14) | 63 |
|  | ***pfmc-2tm*** | 1.81 | 50–0.005 | 1 | 1.81 (±0.10) | 68 |
| **Isolate #4** | ***fructose-bisphospate aldolase*** | 2.02 (±0.01) | 50–0.25 | 2 | n.d. | |
|  | ***var*** | n.d. | | | 1.88 (±0.19) | 46 |
|  | ***rif-A*** |  |  |  | 1.72 (±0.07) | 80 |
|  | ***rif-B*** | 1.57 | 50–0.25 | 1 | 1.64 (±0.06) | 78 |
|  | ***stevor*** | n.d. | | | 1.90 (±0.09) | 64 |
|  | ***pfmc-2tm*** | 1.89 | 50–0.05 | 1 | 1.81 (±0.06) | 63 |
| **Mean** | ***fructose-bisphospate aldolase*** | 1.99 (±0.07) | 75–0.005 | 21 | 1.92 (±0.16) | 1058 |
|  | ***var*** | 1.82 (±0.09) | 50–0.5 | 2 | 1.88 (±0.18) | 237 |
|  | ***rif-A*** | 1.74 | 50–0.05 | 1 | 1.75 (±0.10) | 341 |
|  | ***rif-B*** | 1.56 (±0.05) | 100–0.05 | 7 | 1.61 (±0.09) | 102 |
|  | ***stevor*** | 1.88 (±0.04) | 50–0.0005 | 7 | 1.92 (±0.11) | 330 |
|  | ***pfmc-2tm*** | 1.85 (±0.04) | 50–0.0005 | 9 | 1.83 (±0.10) | 351 |

^1^ Concentration range of template.

^2^ Number of template dilutions.

^3^ Number of samples.

n.d., not determined.
