# Supplementary material for: Temporal Expression and Localization Patterns of Variant Surface Antigens in Clinical Plasmodium falciparum Isolates during Erythrocyte Schizogony
Source: PLoS One. 2012 Nov 15;7(11):e49540. doi: 10.1371/journal.pone.0049540 (PMC3499489; doi:10.1371/journal.pone.0049540)
Supplement: Table S3 — Transcripts and sequences of rif-A , rif-B , stevor and pfmc-2tm genes in P. falciparum isolates #1, #2, #3 and #4 and strain 3D7 during the two peaks of expression in ring and trophozoite stages. (DOC) [file pone.0049540.s014.doc]

**Table S3: Transcripts of *rif-A*, *rif-B*, *stevor* and *pfmc-2tm* genes in *P. falciparum* isolates #1, #2, #3 and #4 and strain 3D7 during the two peaks of expression in ring and trophozoite stages.**

| **Isolate no.** | **MSP1 genotype** | **Parasite stage/sample** | **Sequence (no.) and frequency (%)** | | | | | | | | | | | |
| --- | --- | --- | --- | --- | --- | --- | --- | --- | --- | --- | --- | --- | --- | --- |
|  |  |  | ***rif-A*** | | | ***rif-B*** | | | ***stevor*** | | | ***pfmc-2tm*** | | |
|  |  |  | **N** | **Gene no.** | **%** | **N** | **Gene no.** | **%** | **N** | **Gene no.** | **%** | **N** | **Gene no.** | **%** |
| **1** | Mad20-195 | Ring | 12 | 1 | 25.0 |  |  |  | 14 | 1 | 71.4 | 13 | 1 | 30.8 |
|  | K1-221 | *ex vivo* |  | 2 | 16.7 |  |  |  |  | 2 | 28.6 |  | 2 | 23.1 |
|  |  |  |  | 3-9 | 8.3^#^ |  |  |  |  |  |  |  | 3 | 15.4 |
|  |  |  |  |  |  |  |  |  |  |  |  |  | 4-7 | 7.7^#^ |
|  |  | Trophozoite | 13 | 3 | 23.1 |  |  |  | 13 | 1 | 61.5 | 15 | 2 | 40 |
|  |  | 32 h |  | 4, 10 | 15.4^#^ |  |  |  |  | 3 | 30.8 |  | 1, 3 | 20^#^ |
|  |  |  |  | 5, 11-15 | 7.7^#^ |  |  |  |  | 4 | 7.7 |  | 4, 8, 9 | 6.7^#^ |
|  |  | gDNA | 14 | 16*** | 35.7 | 14 | 1 | 14.3 | 15 | 5* | 20.0 | 13 | 8** | 30.8 |
|  |  |  |  | 6*, 17* | 14.3^#^ |  | 2-13 | 7.1^#^ |  | 2, 4, 6-15 | 6.7^#^ |  | 2 | 23.1 |
|  |  |  |  | 3, 5, 7, 10, 18 | 7.1^#^ |  |  |  |  |  |  |  | 7 | 15.4 |
|  |  |  |  |  |  |  |  |  |  |  |  |  | 1, 4, 10, 11 | 7.7^#^ |
| **2** | RO33-132 | Ring I | 12 | 1, 2 | 16.7^#^ | n.d. |  |  | 13 | 1 | 23.1 | 14 | 1, 2 | 21.4^#^ |
|  | Mad20-212 | *ex vivo* |  | 3-10 | 8.3^#^ |  |  |  |  | 2-4 | 15.4^#^ |  | 3, 4 | 14.3^#^ |
|  |  |  |  |  |  |  |  |  |  | 5-8 | 7.7^#^ |  | 5-8 | 7.1^#^ |
|  |  | Trophozoite | 12 | 3 | 33.3 | n.d. |  |  | 14 | 2 | 42.9 | 14 | 1, 3 | 21.4^#^ |
|  |  | 28 h |  | 11 | 25.0 |  |  |  |  | 8 | 14.3 |  | 2, 9 | 14.3^#^ |
|  |  |  |  | 12 | 16.7 |  |  |  |  | 6, 7, 9-12 | 7.1^#^ |  | 4-7 | 7.1^#^ |
|  |  |  |  | 4, 13, 14 | 8.3^#^ |  |  |  |  |  |  |  |  |  |
|  |  | Schizont | 15 | 3 | 26.7 | n.d. |  |  | n.d. |  |  | n.d. |  |  |
|  |  | 40 h |  | 1 | 20 |  |  |  |  |  |  |  |  |  |
|  |  |  |  | 4, 11-17 | 6.7^#^ |  |  |  |  |  |  |  |  |  |
|  |  | Ring II | 13 | 3, 18 | 15.4^#^ | n.d. |  |  | 14 | 2 | 35.7 | 13 | 3 | 23.1 |
|  |  | 56 h |  | 1, 2, 5, 13, 14, 19-22 | 7.7^#^ |  |  |  |  | 3 | 21.4 |  | 6, 9 | 15.4^#^ |
|  |  |  |  |  |  |  |  |  |  | 13 | 14.3 |  | 1, 2, 5, 7, 10, 11 | 7.7^#^ |
|  |  |  |  |  |  |  |  |  |  | 1, 14-16 | 7.1^#^ |  |  |  |
|  |  | gDNA | 13 | 23*** | 23.1 | 16 | 1* | 18.8 | 15 | 15* | 26.7 | 14 | 11 | 14.3 |
|  |  |  |  | 4*, 19* | 15.4^#^ |  | 2, 3 | 12.5^#^ |  | 2, 4, 6, 12, 14, 16-21 | 6.7^#^ |  | 1, 3, 5-9. 12-16 | 7.1^#^ |
|  |  |  |  | 1, 6, 12, 13, 24, 25 | 7.7^#^ |  | 4-12 | 6.3^#^ |  |  |  |  |  |  |
| **3** | K1-158 | Ring I | 12 | 1-3 | 16.7^#^ | n.d. |  |  | 15 | 1 | 60.0 | 13 | 1 | 38.5 |
|  |  | *ex vivo* |  | 4-9 | 8.3^#^ |  |  |  |  | 2-7 | 6.7^#^ |  | 2 | 23.1 |
|  |  |  |  |  |  |  |  |  |  |  |  |  | 3 | 15.4 |
|  |  |  |  |  |  |  |  |  |  |  |  |  | 4-6 | 7.7^#^ |
|  |  | Trophozoite | 13 | 1 | 38.5 | n.d. |  |  | 15 | 1 | 53.3 | 15 | 6 | 73.3 |
|  |  | 24 h |  | 10 | 15.4 |  |  |  |  | 2 | 20.0 |  | 7 | 13.3 |
|  |  |  |  | 2, 4, 5, 11-13 | 7.7^#^ |  |  |  |  | 3 | 13.3 |  | 2, 4 | 6.7^#^ |
|  |  |  |  |  |  |  |  |  |  | 8, 9 | 6.7^#^ |  |  |  |
|  |  | Schizont | 14 | 2 | 35.7 | n.d. |  |  | n.d. |  |  | n.d. |  |  |
|  |  | 40 h |  | 1 | 21.4 |  |  |  |  |  |  |  |  |  |
|  |  |  |  | 14-19 | 7.1^#^ |  |  |  |  |  |  |  |  |  |
|  |  |  |  |  |  |  |  |  |  |  |  |  |  |  |
|  |  | Ring II | 13 | 1 | 46.2 | n.d. |  |  | 15 | 1 | 53.3 | 15 | 6 | 53.3 |
|  |  | 56 h |  | 10, 20 | 15.4 |  |  |  |  | 10 | 26.7 |  | 7 | 20.0 |
|  |  |  |  | 4, 11, 12 | 7.7^#^ |  |  |  |  | 4, 11, 12 | 6.7^#^ |  | 2 | 13.3 |
|  |  |  |  |  |  |  |  |  |  |  |  |  | 1, 3 | 6.7^#^ |
|  |  | gDNA | 15 | 11, 21-23 | 13.3^#^ | 13 | 1-3 | 15.4^#^ | 15 | 6 | 20 | 14 | 6* | 35.7 |
|  |  |  |  | 1, 3, 4, 12, 14, 24, 25 | 6.7^#^ |  | 4-10 | 7.7^#^ |  | 8, 11 | 13.3^#^ |  | 5 | 21.4 |
|  |  |  |  |  |  |  |  |  |  | 1, 7, 9, 13-17 | 6.7^#^ |  | 2 | 14.3 |
|  |  |  |  |  |  |  |  |  |  |  |  |  | 1, 3, 4, 7 | 7.1^#^ |
| **4** | RO33-125 | Ring I | 17 | 1, 2 | 17.7^#^ | 15 | 1 | 33.3 | 15 | 1 | 40.0 | 17 | 1 | 58.8 |
|  | RO33-132 | 4 h |  | 3-6 | 11.8^#^ |  | 2-11 | 6.7^#^ |  | 2 | 33.3 |  | 2, 3 | 11.8^#^ |
|  | Mad20-183 |  |  | 7-9 | 5.9^#^ |  |  |  |  | 3 | 20.0 |  | 4-6 | 5.9^#^ |
|  | Mad20-193 |  |  |  |  |  |  |  |  | 4 | 6.7 |  |  |  |
|  | Mad20-203 | Trophozoite | 14 | 1, 10 | 35.7^#^ | 17 | 2, 6 | 17.7^#^ | 15 | 1 | 20.0 | 16 | 1 | 25.0 |
|  | K1-158 | 24 h |  | 3, 11-13 | 7.1^#^ |  | 12 | 11.8 |  | 2 | 21.4 |  | 7 | 18.8 |
|  |  |  |  |  |  |  | 1, 3, 5, 7, 8, 13-16 | 5.9^#^ |  | 3 | 14.3 |  | 2, 8, 9 | 12.5^#^ |
|  |  |  |  |  |  |  |  |  |  | 5, 6 | 7.1^#^ |  | 4, 10, 11 | 6.3^#^ |
|  |  | Schizont | n.d. |  |  | 18 | 4 | 38.9 | n.d. |  |  | n.d. |  |  |
|  |  | 40 h |  |  |  |  | 5, 17-19 | 11.1^#^ |  |  |  |  |  |  |
|  |  |  |  |  |  |  | 2, 20, 21 | 5.6^#^ |  |  |  |  |  |  |
|  |  | Ring II | 14 | 3, 10, 14 | 14.3^#^ | 15 | 1 | 20 | 20 | 2 | 35.0 | 19 | 1 | 57.9 |
|  |  | 56-60 h |  | 1, 2, 4, 11, 15-18 | 7.1 |  | 4, 7, 22 | 13.3^#^ |  | 1 | 30.0 |  | 2, 12 | 10.5^#^ |
|  |  |  |  |  |  |  | 3, 5, 6, 17, 23, 24 | 6.7^#^ |  | 3, 7 | 10.0^#^ |  | 5, 13-15 | 5.3^#^ |
|  |  |  |  |  |  |  |  |  |  | 8-10 | 5.0^#^ |  |  |  |
|  |  | gDNA | 14 | 19**, 20** | 14.3^#^ | 16 | 5* | 12.5 | 14 | 11***, 12*** | 21.4^#^ | 14 | 6** | 21.4 |
|  |  |  |  | 1, 2, 5, 10, 11, 21-25 | 7.1^#^ |  | 4, 7, 20, 22-32 | 6.3^#^ |  | 13* | 14.3 |  | 13, 16 | 14.3^#^ |
|  |  |  |  |  |  |  |  |  |  | 2, 14-18 | 7.1^#^ |  | 1, 8, 10, 14, 17-19 | 7.1^#^ |
| **3D7** | K1-203 | Ring-Bio1 | 11 | PFL0010c | 45.5 | 14 | PFI0025c | 85.7 | 14 | PF11_0516 | 35.7 | 14 | PFF0060w | 64.3 |
|  |  | 2.5-10.5 hpi |  | PFC0040w/PF11_0010/PFF1560c | 18.2 |  | PF10_0006 | 7.1 |  | PFD0065w | 28.6 |  | PFA0680c | 35.7 |
|  |  |  |  | PFI0010c | 9.1 |  | PFI1810w | 7.1 |  | PFI0080w | 14.3 |  |  |  |
|  |  |  |  | PFI0030c | 9.1 |  |  |  |  | PFF0030c | 14.3 |  |  |  |
|  |  |  |  | PFB1050w | 9.1 |  |  |  |  | PFC1105w | 7.1 |  |  |  |
|  |  |  |  | PFF1590w | 9.1 |  |  |  |  |  |  |  |  |  |
|  |  | Trophozoite-Bio1 | 10 | PF07_0138/PFI0010c/PFD0025w | 30.0 | n.d. |  |  | 14 | PFF1550w | 28.6 | 11 | PFF0060w | 45.5 |
|  |  | 22.5 hpi |  | PFC0040w/PF11_0010/PFF1560c | 20.0 |  |  |  |  | PF10_0395 | 28.6 |  | PFF1525c | 36.4 |
|  |  |  |  | PFI0010c | 10.0 |  |  |  |  | PF11_0516 | 7.1 |  | PFA0680c | 9.1 |
|  |  |  |  | PFA0045c/PFA0050c/PF08_0138/PFI0010c | 10.0 |  |  |  |  | PFC1105w | 7.1 |  | PF11_0025 | 9.1 |
|  |  |  |  | MAL7P1.216 | 10.0 |  |  |  |  | PF14_0771 | 7.1 |  |  |  |
|  |  |  |  | MAL7P1.185 | 10.0 |  |  |  |  | MAL7P1.218 | 7.1 |  |  |  |
|  |  |  |  | PFL2615w | 10.0 |  |  |  |  | PFL2610w | 7.1 |  |  |  |
|  |  |  |  |  |  |  |  |  |  | PFF0850c | 7.1 |  |  |  |
|  |  | Schizont-Bio1 | 13 | PFI0010c | 38.5 | 14 | PFI0025c | 92.9 | n.d. |  |  | n.d. |  |  |
|  |  | 38.5-42.5 hpi |  | PFB1050w | 15.4 |  | PFA0030c | 7.1 |  |  |  |  |  |  |
|  |  |  |  | PF07_0138/PFI0010c/PFD0025w | 15.4 |  |  |  |  |  |  |  |  |  |
|  |  |  |  | PFA0020w | 15.4 |  |  |  |  |  |  |  |  |  |
|  |  |  |  | PFC0010c | 7.7 |  |  |  |  |  |  |  |  |  |
|  |  |  |  | PFI0075w | 7.7 |  |  |  |  |  |  |  |  |  |
|  |  | Ring-Bio2 | 19 | PFL2615w | 36.8 | 7 | PFI0025c | 42.9 | 14 | PFI0080w | 14.3 | 15 | PFA0680c | 66.7 |
|  |  | 10.5-14.5 hpi |  | PFD1240w/PFB1050w | 21.1 |  | PFA0030c | 28.6 |  | PFB0025c | 14.3 |  | PFF0060w | 26.7 |
|  |  |  |  | PFC1115w | 10.5 |  | PFI1810w | 14.3 |  | PFC0065w | 14.3 |  | PFF1525c | 6.7 |
|  |  |  |  | PFI0010c | 5.3 |  | PFL2655w | 14.3 |  | PFL2610w | 7.1 |  |  |  |
|  |  |  |  | PFC0010c | 5.3 |  |  |  |  | PFI0045c | 7.1 |  |  |  |
|  |  |  |  | PFB1035c | 5.3 |  |  |  |  | PFD0035c | 7.1 |  |  |  |
|  |  |  |  | PFD0040c | 5.3 |  |  |  |  | PF14_0767 | 7.1 |  |  |  |
|  |  |  |  | MAL13P1.4 | 5.3 |  |  |  |  | PFL2620w | 7.1 |  |  |  |
|  |  |  |  | PF11_0529 | 5.3 |  |  |  |  | PF07_0130 | 7.1 |  |  |  |
|  |  |  |  |  |  |  |  |  |  | PFE0030c | 7.1 |  |  |  |
|  |  |  |  |  |  |  |  |  |  | PFC0025c | 7.1 |  |  |  |
|  |  | Trophozoite-Bio2 | 9 | PFL0010c | 11.1 | 21 | PF10_0006 | 23.8 | 15 | PFC1105w | 33.3 | 16 | PFA0680c | 31.3 |
|  |  | 18.5-22.5 hpi |  | PFI0010c | 11.1 |  | PFI0025c | 19.1 |  | PFF1550w | 13.3 |  | PF10_0390 | 31.3 |
|  |  |  |  | PFA0020w | 11.1 |  | PFE1630w | 14.3 |  | PF10_0395 | 13.3 |  | PFF1525c | 12.5 |
|  |  |  |  | PFD1240w/PFB1050w | 11.1 |  | MAL13P1.495 | 9.5 |  | PF14_0767 | 13.3 |  | PFA0065c | 12.5 |
|  |  |  |  | PFC1115w | 11.1 |  | PFA0030c | 4.8 |  | PF07_0130 | 13.3 |  | PFF0060w | 6.3 |
|  |  |  |  | PF14_0006 | 11.1 |  | PFF1545w | 4.8 |  | PFD0065w | 6.7 |  | PFC1080c | 6.3 |
|  |  |  |  | MAL8P1.208/PF08_0142/PFD0015c | 11.1 |  | MAL7P1.215/PF11_0020 | 4.8 |  | PFL2610w | 6.7 |  |  |  |
|  |  |  |  | PFL0025c | 11.1 |  | PF11_0515 | 4.8 |  |  |  |  |  |  |
|  |  |  |  | PFD0030c | 11.1 |  | PFF0005c | 4.8 |  |  |  |  |  |  |
|  |  |  |  |  |  |  | PF14_0005 | 4.8 |  |  |  |  |  |  |
|  |  |  |  |  |  |  | PF10_0394 | 4.8 |  |  |  |  |  |  |
|  |  | Schizont-Bio2 | 14 | PFD1240w/PFB1050w | 42.9 | 24 | PFI0025c | 54.2 | n.d. |  |  | n.d. |  |  |
|  |  | 38.5-42.5 hpi |  | PFL2615w | 14.3 |  | PF10_0006 | 20.8 |  |  |  |  |  |  |
|  |  |  |  | PFI0020w | 14.3 |  | PF14_0003 | 8.3 |  |  |  |  |  |  |
|  |  |  |  | MAL13P1.500 | 7.1 |  | PFI1810w | 4.2 |  |  |  |  |  |  |
|  |  |  |  | PFC1115c | 7.1 |  | PFA0030c | 4.2 |  |  |  |  |  |  |
|  |  |  |  | PFF0035c | 7.1 |  | MAL13P1.495 | 4.2 |  |  |  |  |  |  |
|  |  |  |  | PFD1230c | 7.1 |  | PF13_0006 | 4.2 |  |  |  |  |  |  |
|  |  | gDNA | 20 | PFI0075w | 10.0 | 19 | PF10_0006 | 10.5 | 48 | PFI0045c** | 16.7 | 23 | PF10_0390 | 21.7 |
|  |  |  |  | MAL13P1.535 | 10.0 |  | PFF1570w | 10.5 |  | PFD0035c | 10.4 |  | PFF1525c | 17.4 |
|  |  |  |  | PF10_0398 | 10.0 |  | PF14_0770 | 10.5 |  | MAL7P1.218 | 8.3 |  | PFF0060w | 13.0 |
|  |  |  |  | PFC0040w/PF11_0010/PFF1560w | 5.0 |  | PFF0045c | 10.5 |  | PF14_0767 | 8.3 |  | PF11_0025 | 13.0 |
|  |  |  |  | PFB1050w | 5.0 |  | PFE0025c | 10.5 |  | PFD0065w | 6.3 |  | PFB0985c | 13.0 |
|  |  |  |  | PFF1590w | 5.0 |  | PFI0025c | 5.3 |  | PFL2620w | 6.3 |  | PFC1080c | 13.0 |
|  |  |  |  | PFA0045c/PFA0050c/PF08_0138/PFI0010c | 5.0 |  | PFI1810w | 5.3 |  | PFB0065w | 6.3 |  | PFA0680c | 4.4 |
|  |  |  |  | PFA0040w | 5.0 |  | PFA0030c | 5.3 |  | PF11_0516 | 4.2 |  | PFA0065w | 4.4 |
|  |  |  |  | PF13_0004/PF11_0009 | 5.0 |  | PFB0055c | 5.3 |  | PFL2610w | 4.2 |  |  |  |
|  |  |  |  | PF10_0005 | 5.0 |  | PF14_0003 | 5.3 |  | PF07_0130 | 4.2 |  |  |  |
|  |  |  |  | PF08_0139 | 5.0 |  | PFF1545w | 5.3 |  | PFA0090c | 4.2 |  |  |  |
|  |  |  |  | MAL8P1.219 | 5.0 |  | PF11_0020 | 5.3 |  | PFC1105w | 2.1 |  |  |  |
|  |  |  |  | MAL7P1.52 | 5.0 |  | MAL7P1.215/PF11_0020 | 5.3 |  | PF10_0395 | 2.1 |  |  |  |
|  |  |  |  | MAL7P1.217 | 5.0 |  | MAL7P1.200 | 5.3 |  | PF14_0771 | 2.1 |  |  |  |
|  |  |  |  | PFI1815c | 5.0 |  |  |  |  | PFF0850c | 2.1 |  |  |  |
|  |  |  |  | MAL13P1.500 | 5.0 |  |  |  |  | PFA0750w | 2.1 |  |  |  |
|  |  |  |  | PFI0065w | 5.0 |  |  |  |  | PFB0025c | 2.1 |  |  |  |
|  |  |  |  |  |  |  |  |  |  | PFE0030c | 2.1 |  |  |  |
|  |  |  |  |  |  |  |  |  |  | PFB0050c | 2.1 |  |  |  |
|  |  |  |  |  |  |  |  |  |  | MAL7P1.223 | 2.1 |  |  |  |
|  |  |  |  |  |  |  |  |  |  | PF14_0007 | 2.1 |  |  |  |

^#^ Genes indicated were found with identical frequency.

* P_c_ < 0.05.

** P_c_ < 0.01.

*** P_c_ < 0.001.

h, hours of *in vitro* cultivation.

hpi, hours post-infection.

N, number of clones sequenced.

n.d., not determined.
