# Supplementary material for: Temporal Expression and Localization Patterns of Variant Surface Antigens in Clinical Plasmodium falciparum Isolates during Erythrocyte Schizogony
Source: PLoS One. 2012 Nov 15;7(11):e49540. doi: 10.1371/journal.pone.0049540 (PMC3499489; doi:10.1371/journal.pone.0049540)
Supplement: Table S4 — Patient and isolate characteristics. (DOC) [file pone.0049540.s015.doc]

|  | **Sample** | | | | |
| --- | --- | --- | --- | --- | --- |
|  | **#1** | **#2** | **#3** | **#4** | **#5** |
| **Patient characteristics** |  |  |  |  |  |
| Age, years | 52 | 49 | 49 | 29 | 58 |
| Sex | Male | Male | Male | Male | Male |
| Parasitemia, % | 1 | 2 | 1.5 | 3 | 36 |
| Native country | Ghana | Germany | Germany | Ghana | Germany |
| National residence | Germany | Germany | France | Germany | Germany |
| Sample volume, mL | 20 | 30 | 30 | 30 | 3 |
| **Isolate characteristics** |  |  |  |  |  |
| Origin | Ghana | Gambia | Senegal | Ghana | Togo |
| MSP1 genotypes, bp | Mad20-195  K1-221 | RO33-132  Mad20-212 | K1-158 | RO33-125  RO33-132  Mad20-183  Mad20-193  Mad20-203  K1-158 | Mad20-208  Mad20-221 |

**Table S4: Patient and isolate characteristics.**
